# A New Useful Exponential Model with Applications to Quality Control and Actuarial Data

**DOI:** 10.1155/2022/2489998

**Published:** 2022-06-09

**Authors:** Aisha Fayomi, M. H. Tahir, Ali Algarni, M. Imran, Farrukh Jamal

**Affiliations:** ^1^Faculty of Science, Department of Statistics, King Abdulaziz University, Jeddah, Saudi Arabia; ^2^Department of Statistics, Faculty of Computing, The Islamia University of Bahawalpur, Bahawalpur 63100, Pakistan

## Abstract

The compounding approach is used to introduce a new family of distributions called *exponentiated Bell G*, analogy to *exponentiated G Poisson*. Several essential properties of the proposed family are obtained. The special model called exponentiated Bell exponential (EBellE) is presented along with properties. Furthermore, the risk theory related measures including value-at-risk and expected-shortfall are also computed for the special model. Group acceptance sampling plan is designed when a lifetime of a product or item follows an EBellE model taking median as a quality parameter. The parameters of the proposed model are estimated by considering maximum likelihood approach along with simulation analysis. The usefulness of the proposed model is illustrated by practical means which yield better fits as compared to several exponential related extended models.

## 1. Introduction

Effective implementation of mathematical and statistical models enables the actuarial scientists to know as much as possible about future claims in a portfolio. These models serve as a guide to achieve better business and risk management decision and policies. Actuaries usually deal with a complex data such as right skewed, unimodal, and having heavy tail. The readers are referred to works of Klugman et al. [1], Cooray and Ananda et al. [2], Lane [3], Vernic [4], and Ibragimov et al. [5]. At the same time, they are eager on some flexible models which are capable of capturing the behaviours of such data to finding along with information when the real development deviates from the expected. The classical models are limited with their tail properties and goodness of fit tests. For instance, Pareto, Lomax, Fisk, and Dagum distribution are excessively used to model statistical size distributions in economics and actuarial sciences but often failed to provide better fits for many application. The Weibull distribution is appropriate for small losses but fail to uncover adequate trend, level, and trajectory for large losses [6]. The reader are referred to [7] for detail discussion on statistical size distributions which can be used in economics and actuarial sciences. To overcome the drawback of classical models, a substantial progress on persistent base related to distribution theory is documented in statistical literature. From the last couple of decades, the emerging trend has been seen in the generalization of the existing classical models. The models are extended by adopting different modes of adding one or more additional shape parameter(s) in the distribution. The basic aim of this whole exercise is to improve the tail properties as well as goodness of fit test of the classical models. There are several well-known generators which are documented in the statistical literature; the readers are referred to the works of Tahir and Nadarajah [8], Tahir and Cordeiro [9], Maurya and Nadarajah [10], and Lee et al. [11].

Several new models related to claim data have recently been reported in statistical literature. Ahmad et al. [12] proposed a new method to define heavy-tailed distributions called the exponentiated power Weibull distribution with application to medical care insurance and vehicle insurance. Calderin–Ojeda and Kwok [13] presented a new class of composite model by using the Stoppa distribution and mode matching procedure and modelling the actuarial claims data of mixed sizes. Ahmad et al. [14] suggested nine new methods to define new distributions suitable for modelling heavy right-tail data with application to medical care insurance and vehicle insurance. Afify et al. [15] proposed a new heavy-tailed exponential distribution with application to unemployment claim data. Ahmad et al. [16] introduced a class of claim distributions useful in a number of lifetime analyses. A special submodel of the proposed family, called the Weibull claim model, is considered in detail with claim data application. Among classical discrete distributions, Poisson distribution is a most frequently used distribution for count data. Furthermore, it is extended into G-class and several transformation and family of distributions have been proposed. A detail review study on Poisson generated family of distributions, extensions, and transformation is recently presented by [10]. Castellares et al. [17] introduced a discrete Bell distribution from well-known Bell numbers, as a competitor or counterpart to Poisson distribution which exhibits many interesting properties such as a single parameter distribution, and it belongs to one-parameter exponential family of distributions and the Poisson distributions. They investigated that the Poisson model cannot be nested into the Bell model, but small values of the parameter the Bell model tends to Poisson distribution. Furthermore, the Bell model is infinity divisible and has larger variance as compared to the mean, which can be used to overcome the phenomenon of over-dispersion and zero-vertex for count data. The characteristics of the Bell model motivated us to develop a generalized class of distributions through compounding and to compare its mathematical and empirical characteristics with compounded Poisson-G class and its special models.

The rest of the study is organized as follows. In Section 2, we define the proposed EBell-G family of distributions.  Section 3 provides the general mathematical and structural properties of EBell-G family of distributions including linear representation of density, quantile function, *r*th moments, probability weighted moments, analytical shapes of the density and hazard rate, entropy measures, reversed order statistics, upper record statistics, stochastic ordering, and parameters' estimation by using maximum likelihood estimation.  Section 4 illustrates the layout of the special model called EBellE as well as its essential properties, while Section 5 shows the commonly used actuarial measures, specially value-at-risk and expected-shortfall.  Section 6 are illustrated group acceptance sampling plane when a lifetime of a certain product or item follows the EBellE model which is presented. The simulation analysis is presented in Section 7, and Section 8 contains the application of real datasets. The concluding remarks are given in Section 9.

## 2. Layout and Formulation of EBell-G Family

A single parameter discrete Bell distribution has been recently introduced by Castellares et al. [17], which is an analogy to discrete Poisson distribution but provides better fits compared to other discrete models including the Poisson model. The following expression given by Bell [18] is

(1) $$\begin{array}{c} \exp\left[e^{x}-1\right]=\sum_{n=0}^{\infty }\frac{B_{n}}{n!}x^{n}, \end{array}$$

where *B*_*n*_ denote the Bell numbers and can be derived from the following mathematical expression:

(2) $$\begin{array}{c} B_{n}=\frac{1}{e}\sum_{k=0}^{\infty }\frac{k^{n}}{k!}. \end{array}$$

(3) $$\begin{array}{c} \mathbb{P}r\left(X=x\right)=\frac{\lambda^{n}e^{1-e^{\lambda }}B_{n}}{n!\left[1-e^{1-e^{\lambda }}\right]},\;x=1,2\ldots . \end{array}$$

**Remark 1 .**

The Bell number *B*_*n*_ in (2) is the *n*th moment of the Poisson distribution with parameter equal to 1.

By considering equations (1) and (2), Castellares et al. [17] introduced a single-parameter Bell distribution defined by the following probability mass function (pmf) as

(4) $$\begin{array}{c} \mathbb{P}r\left(X=x\right)=\frac{\lambda^{x}e^{-e^{\lambda }+1}B_{x}}{x!},\;x=0,1,2\ldots . \end{array}$$

**Proposition 1 .**

*LetXfollow a discrete Bell model with parameterλ; then, the following expression represents the pmf of Bell truncated model as*

We first give the motivation for the proposed family. Suppose a system is having *N* subsystems that are working or functioning independently at a given specific time. Here, *Y*_*i*_ denotes the life of *ith* subsystem and *θ* parallel units constitutes the subsystem. Furthermore, the system will fail or remain functioning if all the subsystem fail; this is for the parallel system. On the contrary, for series system, the failure of any subsystem yields complete destruction of the whole system. Let us have a random variable (*rv*)*N* that follows any discrete distribution having pmf ℙ(*N*=*n*). Here, we suppose that a component *Z*_*i*,1_,……*Z*_*i*,*θ*_ having failure time for the *ith* subsystem are i.i.d. with suitable cdf depending upon the vector *τ*, say for *X* > 0, *T*[*G*(*x*, *τ*), *θ*]=*G*(*x*, *τ*)^*θ*^. If we define *Y*=min{*Y*_1_,……*Y*_*N*_}, then the conditional cdf of *Y* given *N* is as follows:

(5) $$\begin{array}{c} F\left(Y|N\right)=P\left[\min\left(Y_{1},\ldots ,Y_{N}\right)<y|N\right]=1-{\left[1-P^{\theta }\left(Z_{1,1}\leq y\right)\right]}^{N}\\ =1-{\left[1-G{\left(y;\tau \right)}^{\theta }\right]}^{N}. \end{array}$$

The unconditional cdf of *Y* corresponding to (5) is given by

(6) $$\begin{array}{c} F\left(y\right)=\overset{\infty }{\underset{n=1}{\sum }}F\left(y|N\right)P\left(N=n\right)=1-{\left[1-G{\left(y;\tau \right)}^{\theta }\right]}^{N}P\left(N=n\right). \end{array}$$

By using the Bell truncated model given in Eq. (4) and then using Eq. (6), the unconditional cdf of *X* is defined below as follows.

(7) $$\begin{array}{c} F\left(x\right)=\frac{1-\exp\left\{-e^{\lambda }\left[1-e^{-\lambda G^{\theta }\left(x\right)}\right]\right\}}{\left\{1-\exp\left[1-e^{\lambda }\right]\right\}}, \end{array}$$

(8) $$\begin{array}{c} f\left(x\right)=\frac{\lambda \theta g\left(x\right)G^{\theta -1}\left(x\right)\exp\left\{\lambda \left[1-G^{\theta }\left(x\right)\right]\right\}\exp\left\{-e^{\lambda }\left[1-e^{-\lambda G^{\theta }\left(x\right)}\right]\right\}}{\left\{1-\exp\left[1-e^{\lambda }\right]\right\}}, \end{array}$$

(9) $$\begin{array}{c} s\left(x\right)=\frac{\exp\left\{-e^{\lambda }\left[1-e^{-\lambda G^{\theta }\left(x\right)}\right]\right\}-\exp\left[1-e^{\lambda }\right]}{\left\{1-\exp\left[1-e^{\lambda }\right]\right\}}, \end{array}$$

**Proposition 2 .**

*LetX*∼*EBell-G*(*λ*, *θ*, *ξ*), *forx* > 0*andλ*, *θ*, *ξ* > 0; *then, its cumulative distribution function (cdf) having baseline pdf and cdf respectivelyg*(*x*)*andG*(*x*)*is given by*

**Proposition 3 .**

*LetX*∼*EBell-G*(*λ*, *θ*, *ξ*)*forx* > 0*andλ*, *θ*, *ξ* > 0; *then, its probability distribution function (pdf) having Eq.* (8)*, with baseline pdf and cdf respectivelyg*(*x*)*andG*(*x*), *is given by*

**Proposition 4 .**

*LetX*∼*EBell-G*(*λ*, *θ*, *ξ*)*forx* > 0*andλ*, *θ*, *ξ* > 0; *then, its survival function (sf) and hazard rate function (hrf) are, respectively, given by*

## 3. Properties of the EBell-G Family

This section provides some mathematical properties of the EBell-G family of distributions.

### 3.1. Quantile Function

Quantile function (qf) is an important measure for generating random numbers and several other important uses in quality control sampling plans and in risk theory; the two important commonly used measures value-at-risk (VaR) and expected-shortfall (ES) which depend on qf and is given as follows.

(10) $$\begin{array}{c} Q\left(u\right)=F^{-1}{\left\{1-\lambda^{-1}\left[\log\left\{\log\left\{1-u\left\{1-\exp\left[1-e^{\lambda }\right]\right\}\right\}+\exp\left(\lambda \right)\right\}\right]\right\}}^{1/\theta }. \end{array}$$

**Proposition 5 .**

*LetX*∼*EBell-G*(*λ*, *θ*, *ξ*)*forx* > 0*andλ*, *θ*, *ξ* > 0; *then, the expression of qf is given below, whereu* ~ *uniform*(0,1), *and by replacingu*=0.5, *it yields the median of the EBell-G:*

### 3.2. Analytic Shapes of the Density and Hazard Rate Function

The analytical shapes of the density and hrf can be yielded for EBellE, respectively, as follows:

(11) $$\begin{array}{c} \frac{g^{\prime }\left(x\right)}{g\left(x\right)}+\left(\theta -1\right)\frac{g\left(x\right)}{G\left(x\right)}-\lambda \theta g\left(x\right)G^{\theta -1}\left(x\right)\left\{1+\exp\left\{\lambda \left[1-G^{\theta }\left(x\right)\right]\right\}\right\}=0,\\ \frac{g^{\prime }\left(x\right)}{g\left(x\right)}+\left(\theta -1\right)\frac{g\left(x\right)}{G\left(x\right)}-\lambda \theta g\left(x\right)G^{\theta -1}\left(x\right)=0. \end{array}$$

### 3.3. Useful Expansions

Here, we show the useful expansion for EBell-G density can be used to drive several important properties by taking into account the following two series to obtain the expansion for EBell-G.

(12) $$\begin{array}{c} {\left(1-t\right)}^{b}=\sum_{c=0}^{\infty }{\left(-1\right)}^{c}\left(\begin{array}{c} b\\ c \end{array}\right)t^{c}. \end{array}$$

**Proposition 6 .**

*The generalized binomial expansion which holds for any real noninteger b and *|*t*| < 1*is*

The power series for exponential function is given by Bourguignon et al. [19] and is given as follows:

(13) $$\begin{array}{c} \exp\left[-\alpha {\left(x\right)}^{b}\right]=\sum_{k=0}^{\infty }{\left(-1\right)}^{k}\alpha^{k}\frac{x^{kb}}{k!}. \end{array}$$

Therefore, by using Eq. (11) to Eq. (8), we can deduce pdf and cdf, simultaneously, as

(14) $$\begin{array}{c} f\left(x;\lambda ,\theta ,\xi \right)=\sum_{v=0}^{\infty }w_{v}h_{\theta \left(v+1\right)}\left(x\right), \end{array}$$

(15) $$\begin{array}{c} F\left(x;\lambda ,\theta ,\xi \right)=\overset{\infty }{\underset{v=0}{\sum }}w_{v}H_{\theta \left(v+1\right)}\left(x\right), \end{array}$$

where

(16) $$\begin{array}{c} w_{v}=\frac{\lambda^{1+v}{\left(v+1\right)}^{-1}}{\left[1-\exp\left(1-e^{\lambda }\right)\right]v!}\overset{\infty }{\underset{k}{\sum }}\overset{\infty }{\underset{r=0}{\sum }}\frac{1}{k!}{\left(-1\right)}^{k+r+v}\left(\begin{array}{c} k\\ r \end{array}\right){\left(1+r\right)}^{v}e^{\lambda \left(1+k\right)}, \end{array}$$

are constants satisfying ∑_*v*=0_^*∞*^*w*_*v*_=1. Eq. (12) represents exp-G, that is, *h*_*θ*(*v*+1)_(*x*) and the term *θ*(*v*+1) is treated as the power parameter. By using Eq. (12), numerous properties of *G*-class can be obtained.

### 3.4. Mathematical Properties

One can derive some important mathematical properties by considering Eq. (12). The *r*th raw moment of *X* is given by

(17) $$\begin{array}{c} \mu_{r}^{\prime }=\sum_{v=0}^{\infty }w_{v}E\left[X_{\theta \left(v+1\right)}^{r}\right], \end{array}$$

where *E*[*X*_*θ*(*v*+1)_^*r*^] follows a exp-G with *θ*(*v*+1) treated as the power parameter, and by taking *r*=1, in (14), yields the mean for *X*.

The incomplete moments are important and have many practical uses. The expression of *s*th incomplete moments, denoted by *φ*_*s*_(*t*), is defined by *φ*_*s*_(*t*)=∫_−*∞*_^*t*^*x*^*s*^*f*(*x*)d*x* and can be obtained by using Eq. (12) as

(18) $$\begin{array}{c} \varphi_{s}\left(t\right)=\sum_{v=0}^{\infty }w_{v}\int_{-\infty }^{t}x^{s}h_{\theta \left(v+1\right)}\left(x\right)\text{d}x. \end{array}$$

The first incomplete moment of the EBell-G family can be obtained as by taking *s*=1 in Eq. (15). The *s*th incomplete moment is an important to compute several measures, namely, mean deviations from mean and median, mean waiting time, conditional moments, and income inequality measures among others.

### 3.5. Probability Weighted Moments

The (*s*, *r*)th probability weighted moments (PWM) of *X* following the EBell-G family, say *ρ*_*s*,*r*_, is formally defined by

(19) $$\begin{array}{c} \rho_{s,r}=E\left[X^{s}F{\left(x\right)}^{r}\right]=\int_{-\infty }^{+\infty }X^{s}F{\left(x\right)}^{r}f\left(x\right)\text{d}x. \end{array}$$

By using Eq. (7) and Eq. (8), we can obtain

(20) $$\begin{array}{c} \rho_{s,r}=\sum_{Q=0}^{\infty }w_{Q}E\left[Y_{\theta \left(Q+1\right)}^{s}\right], \end{array}$$

where

(21) $$\begin{array}{c} w_{Q}=\frac{{\left(-1\right)}^{Q}\lambda^{1+Q}e^{\lambda }}{Q!\left(Q+1\right){\left[1-\exp\left(1-e^{\lambda }\right)\right]}^{\left(r+1\right)}}\overset{\infty }{\underset{z,v,p=0}{\sum }}{\left(-1\right)}^{z+v+p}\left(\begin{array}{c} r\\ z \end{array}\right)\left(\begin{array}{c} v\\ p \end{array}\right){\left(\left(1+z\right)e^{\lambda }\right)}^{v}\frac{{\left(1+p\right)}^{Q}}{v!}. \end{array}$$

### 3.6. Entropy Measures

The entropy measures are important to underline the randomness or uncertainty or diversity of the system. The most frequently used index of dispersion in ecology as well as in statistics is called the Rényi entropy *I*_*δ*_(*x*) and is defined by the following expression:

(22) $$\begin{array}{c} I_{\delta }\left(x\right)={\left(1-\delta \right)}^{-1}\log\int_{-\infty }^{+\infty }f{\left(x\right)}^{\delta }\text{d}x, \end{array}$$

where *δ* > 0 and *δ* ≠ 1, which then follows

(23) $$\begin{array}{c} {\text{I}}_{\delta }\left(X\right)={\left(1-\delta \right)}^{-1}\log\left[\sum_{b=0}^{\infty }Q_{b}\int_{-\infty }^{+\infty }g{\left(x\right)}^{\delta }G{\left(x\right)}^{\theta \;b+\delta \left(\theta -1\right)}\text{d}x\right], \end{array}$$

where

(24) $$\begin{array}{c} Q_{b}=\frac{\theta^{\delta }\lambda^{\left(\delta +b\right)}e^{\delta \lambda }}{b!{\left[1-\exp\left(1-e^{\lambda }\right)\right]}^{\delta }}\sum_{t,s=0}^{\infty }\frac{1}{t!}{\left(-1\right)}^{t+s+b}\left(\begin{array}{c} t\\ s \end{array}\right){\left(\delta e^{\lambda }\right)}^{t}{\left(s+\delta \right)}^{b}. \end{array}$$

The Shannon entropy say, *H*_*q*_(*x*), can be obtained by the following expression:

(25) $$\begin{array}{c} H_{q}\left(x\right)={\left(1-q\right)}^{-1}\log\left[\sum_{b=0}^{\infty }Q_{b}\int_{-\infty }^{+\infty }g{\left(x\right)}^{q}G{\left(x\right)}^{b}\text{d}x\right], \end{array}$$

where *q* > 0 and *q* ≠ 1 and

(26) $$\begin{array}{c} Q_{b}=\frac{\theta^{q}\lambda^{\left(q+b\right)}e^{q\lambda }}{b!{\left[1-\exp\left(1-e^{\lambda }\right)\right]}^{q}}\sum_{t,s=0}^{\infty }\frac{1}{t!}{\left(-1\right)}^{t+s+b}\left(\begin{array}{c} t\\ s \end{array}\right){\left(qe\right)}^{t}{\left(s+q\right)}^{b}. \end{array}$$

### 3.7. Order Statistics

Here, we derived the explicit expression for the *i*th-order statistics for EBell-G, say *f*_*i*:*n*_(*x*). Let a sample of size be *n*; then, the pdf of *i*th-order statistics is defined by

(27) $$\begin{array}{c} f_{i:n}\left(x\right)=\frac{1}{B\left(i,n-i+1\right)}f\left(x\right)\sum_{l=0}^{n-i}{\left(-1\right)}^{l}\left(\begin{array}{c} n-i\\ l \end{array}\right)F{\left(x\right)}^{i+l-1}. \end{array}$$

By using Eq. (7) and Eq. (8), the density for EBell-G can be written as

(28) $$\begin{array}{c} f_{i:n}\left(x\right)=\sum_{j=0}^{\infty }Q_{i:n}^{\left(j\right)}h_{\theta \left(j+1\right)}\left(x\right), \end{array}$$

where

(29) $$\begin{array}{c} Q_{i:n}^{\left(j\right)}=\frac{\lambda^{\left(1+j\right)}{\left(1+j\right)}^{-1}{\left(-1\right)}^{j}e^{\lambda }}{j!{\left[1-\exp\left(1-e^{\lambda }\right)\right]}^{i+l}}\frac{1}{B\left(i,n-i+1\right)}\overset{n-i}{\underset{l=0}{\sum }}\overset{i+l-1}{\underset{p=0}{\sum }}\overset{\infty }{\underset{z,i}{\sum }}\frac{e^{\lambda z}}{z!}{\left(-1\right)}^{p+z+i+l}\\ \times \left(\begin{array}{c} i+l-1\\ p \end{array}\right)\left(\begin{array}{c} n-i\\ l \end{array}\right)\left(\begin{array}{c} z\\ i \end{array}\right){\left(1+p\right)}^{z}{\left(1+i\right)}^{j}. \end{array}$$

The *s*th moment of order statistic can be obtained as

(30) $$\begin{array}{c} E\left(X_{i:n}^{s}\right)=\sum_{j=0}^{\infty }Q_{i:n}^{\left(j\right)}\mu_{\theta \left(j+1\right)}^{\left(s\right)}, \end{array}$$

where *μ*_*θ*(*j*+1)_^(*s*)^ is the *s*th moment of Exp-G distribution with power parameter *θ*(*j*+1).

### 3.8. Reversed Order Statistics

The reversed order statistics can be used when *x*_1_,……, *x*_*n*_ are arranged in the decreasing order; for more detail, see the work of Jamal et al. [20]. The pdf of *X*_*r*(*re*):*n*_, represented by *f*_*r*(*re*):*n*_(*x*)=*f*_*n*−*r*+1:*n*_(*x*), is defined by

(31) $$\begin{array}{c} f_{r\left(re\right):n}\left(x\right)=C_{r:n}f\left(x\right){\left[F\left(x\right)\right]}^{n-r}{\left[1-F\left(x\right)\right]}^{r-1},\;x\in \mathbb{R}, \end{array}$$

and

(32) $$\begin{array}{c} f_{r\left(re\right):n}\left(x\right)=C_{r:n}f\left(x\right)\sum_{l=0}^{r-1}{\left(-1\right)}^{l}\left(\begin{array}{c} r-1\\ l \end{array}\right){\left[F\left(x\right)\right]}^{n-r+l}. \end{array}$$

Consider

(33) $$\begin{array}{c} I=f\left(x\right)\frac{{\left[1-\exp\left\{-e^{\lambda }\left[1-e^{-\lambda G^{\theta }\left(x\right)}\right]\right\}\right]}^{n-r+l}}{{\left\{1-\exp\left[1-e^{\lambda }\right]\right\}}^{n-r+l}}. \end{array}$$

By using Eq. (10), we can obtain

(34) $$\begin{array}{c} I=\frac{f\left(x\right)}{{\left\{1-\exp\left[1-e^{\lambda }\right]\right\}}^{n-r+l}}\sum_{p=0}^{\left(n-r+l\right)}{\left(-1\right)}^{p}\left(\begin{array}{c} n-r+l\\ p \end{array}\right)\exp\left\{-pe^{\lambda }\left[1-e^{-\lambda G^{\theta }\left(x\right)}\right]\right\}. \end{array}$$

Then, by using Eq. (11), we can have

(35) $$\begin{array}{c} I=\frac{\lambda \theta g\left(x\right)G^{\theta -1}\left(x\right)}{{\left\{1-\exp\left[1-e^{\lambda }\right]\right\}}^{n-r+l+1}}\exp\left\{\lambda \left[1-G^{\theta }\left(x\right)\right]\right\}\sum_{p=0}^{\left(n-r+l\right)}{\left(-1\right)}^{p}\left(\begin{array}{c} n-r+l\\ p \end{array}\right)\\ \times \;\exp\left\{-e^{\lambda }\left[1-e^{-\lambda G^{\theta }\left(x\right)}\right]\left(1+p\right)\right\}. \end{array}$$

Let us consider

(36) $$\begin{array}{c} \exp\left\{-e^{\lambda }\left[1-e^{-\lambda G^{\theta }\left(x\right)}\right]\left(1+p\right)\right\}=\overset{\infty }{\underset{z=0}{\sum }}\frac{{\left(-1\right)}^{z}}{z!}{\left[e^{\lambda }\left(1+p\right)\right]}^{z}{\left[1-e^{-\lambda G^{\theta }\left(x\right)}\right]}^{z}. \end{array}$$

After simplification, we have shapes:

(37) $$\begin{array}{c} I=\frac{\lambda \theta g\left(x\right)G^{\theta -1}\left(x\right)}{{\left\{1-\exp\left[1-e^{\lambda }\right]\right\}}^{n-r+l+1}}\exp\left\{\lambda \left[1-G^{\theta }\left(x\right)\right]\right\}\overset{\left(n-r+l\right)}{\underset{p=0}{\sum }}\overset{\infty }{\underset{z=0}{\sum }}\overset{\infty }{\underset{z=0}{\sum }}{\left(-1\right)}^{p+z+k}\left(\begin{array}{c} n-r+l\\ p \end{array}\right)\\ \times \left(\begin{array}{c} z\\ k \end{array}\right)\frac{{\left[e^{\lambda }\left(1+p\right)\right]}^{z}}{z!}e^{-\lambda kG^{\theta }\left(x\right)},\\ I=\frac{\lambda \theta g\left(x\right)G^{\theta -1}\left(x\right)e^{\lambda }}{{\left\{1-\exp\left[1-e^{\lambda }\right]\right\}}^{n-r+l+1}}\overset{\left(n-r+l\right)}{\underset{p=0}{\sum }}\overset{\infty }{\underset{z=0}{\sum }}\overset{\infty }{\underset{k=0}{\sum }}{\left(-1\right)}^{p+z+k}\left(\begin{array}{c} n-r+l\\ p \end{array}\right)\left(\begin{array}{c} z\\ k \end{array}\right)\\ \times \frac{{\left[e^{\lambda }\left(1+p\right)\right]}^{z}}{z!}e^{-\lambda \left(1+k\right)G^{\theta }\left(x\right)}, \end{array}$$

Finally,

(38) $$\begin{array}{c} I=\frac{\lambda e^{\lambda }}{{\left\{1-\exp\left[1-e^{\lambda }\right]\right\}}^{n-r+l+1}}\overset{\left(n-r+l\right)}{\underset{p=0}{\sum }}\overset{\infty }{\underset{z=0}{\sum }}\overset{\infty }{\underset{k=0}{\sum }}\overset{\infty }{\underset{j=0}{\sum }}{\left(-1\right)}^{p+z+k+j}\left(\begin{array}{c} n-r+l\\ p \end{array}\right)\left(\begin{array}{c} z\\ k \end{array}\right)\frac{{\left[e^{\lambda }\left(1+p\right)\right]}^{z}}{z!j!\left(j+1\right)}\\ \times {\left[\lambda \left(1+k\right)\right]}^{j}\theta \left(j+1\right)g\left(x\right)G{\left(x\right)}^{\theta \left(j+1\right)-1}. \end{array}$$

The reduced form will be

(39) $$\begin{array}{c} f_{r\left(re\right):n}\left(x\right)=\sum_{j=0}^{\infty }W_{r:n}^{\left(j\right)}h_{\theta \left(j+1\right)}\left(x\right), \end{array}$$

where *h*_*θ*(*j*+1)_=*θ*(*j*+1)*g*(*x*)*G*(*x*)^*θ*(*j*+1)−1^ and

(40) $$\begin{array}{c} W_{r:n}^{\left(j\right)}=\frac{\lambda e^{\lambda }}{{\left\{1-\exp\left[1-e^{\lambda }\right]\right\}}^{n-r+l+1}}C_{r:n}\overset{r-1}{\underset{l=0}{\sum }}\overset{\left(n-r+l\right)}{\underset{p=0}{\sum }}\overset{\infty }{\underset{z=0}{\sum }}\overset{\infty }{\underset{k=0}{\sum }}{\left(-1\right)}^{p+z+k+j+l}\left(\begin{array}{c} n-r+l\\ p \end{array}\right)\left(\begin{array}{c} r-1\\ l \end{array}\right)\\ \times \left(\begin{array}{c} z\\ k \end{array}\right)\frac{{\left[e^{\lambda }\left(1+p\right)\right]}^{z}}{z!j!\left(j+1\right)}{\left[\lambda \left(1+k\right)\right]}^{j}. \end{array}$$

The *p*th moment of reversed-order statistic can be obtained as

(41) $$\begin{array}{c} E\left(X_{r:n}^{p}\right)=\sum_{j=0}^{\infty }W_{i:n}^{\left(j\right)}\mu_{\theta \left(j+1\right)}^{\left(p\right)}, \end{array}$$

where *μ*_*θ*(*j*+1)_^(*p*)^ is the *p*th moment of Exp-G distribution with power parameter *θ*(*j*+1).

### 3.9. Upper Record Statistics

Record value is an important measure in many practical areas, for instance, economics data and weather and athletic events. Let us consider (*X*_*n*_)_*n*≥1_ a sequence of independent *rvs* having the same distribution. Let us denote by *F*(*x*) and *f*(*x*) the related cdf and pdf of EBellE distribution, respectively, and *X*_*i*:*n*_ be the *ith*-order statistic as described previously. For fixed *k* ≥ 1, the pdf of *k*th upper record statistic is defined by

(42) $$\begin{array}{c} f_{Y_{n}^{\left(k\right)}}\left(x\right)=\frac{k!}{\left(n-1\right)!}{\left[R\left(x\right)\right]}^{n-1}{\left[1-F\left(x\right)\right]}^{k-1}f\left(x\right), \end{array}$$

where *R*(*x*)=−ln[1 − *F*(*x*)] correspond to the cumulative hazard rate function related to *F*(*x*). Eq. (20) can also be expressed for *R*(*x*)=*e*^*λ*^[1 − *e*^−*λG*^*θ*^(*x*)^], by using (7), as

(43) $$\begin{array}{c} f_{Y_{n}^{\left(k\right)}}\left(x\right)=\frac{k!e^{\lambda \left(n-1\right)}}{\left(n-1\right)!}\sum_{t=0}^{k-1}{\left(-1\right)}^{t}\left(\begin{array}{c} k-1\\ t \end{array}\right){\left[1-e^{-\lambda G\left(x\right)}\right]}^{n-1}f\left(x\right)F{\left(x\right)}^{t}. \end{array}$$

Considering the last terms,

(44) $$\begin{array}{c} I={\left[1-e^{-\lambda G^{\theta }\left(x\right)}\right]}^{n-1}f\left(x\right)F{\left(x\right)}^{t} \end{array}$$

and after using series, we obtain

(45) $$\begin{array}{c} I={\left[1-e^{-\lambda G^{\theta }\left(x\right)}\right]}^{n-1}\frac{\lambda \theta g\left(x\right)G^{\theta -1}\left(x\right)\exp\left\{\lambda \left[1-G^{\theta }\left(x\right)\right]\right\}}{{\left\{1-\exp\left[1-e^{\lambda }\right]\right\}}^{t+1}}\overset{\infty }{\underset{z=0}{\sum }}{\left(-1\right)}^{z}\left(\begin{array}{c} t\\ z \end{array}\right)\\ \times \;\exp\left\{-e^{\lambda }\left[1-e^{-\lambda G^{\theta }\left(x\right)}\right]\left(z+1\right)\right\}, \end{array}$$

Using power series given in Eq. (11), we obtain

(46) $$\begin{array}{c} \exp\left\{-e^{\lambda }\left[1-e^{-\lambda G^{\theta }\left(x\right)}\right]\left(1+z\right)\right\}=\overset{\infty }{\underset{v=0}{\sum }}\frac{{\left(-1\right)}^{v}}{v!}{\left[e^{\lambda }\left(1+z\right)\right]}^{v}{\left[1-e^{-\lambda G^{\theta }\left(x\right)}\right]}^{v}. \end{array}$$

Now, the above expression becomes

(47) $$\begin{array}{c} I=\frac{\lambda \theta g\left(x\right)G^{\theta -1}\left(x\right)\exp\left\{\lambda \left[1-G^{\theta }\left(x\right)\right]\right\}}{{\left\{1-\exp\left[1-e^{\lambda }\right]\right\}}^{t+1}}\overset{\infty }{\underset{z=0}{\sum }}\overset{\infty }{\underset{v=0}{\sum }}\frac{{\left(-1\right)}^{z+v}}{v!}\left(\begin{array}{c} t\\ z \end{array}\right){\left[e^{\lambda }\left(1+z\right)\right]}^{v}\\ \times {\left[1-e^{-\lambda G^{\theta }\left(x\right)}\right]}^{v+n-1}. \end{array}$$

By using Eq. (11) again, we obtain

(48) $$\begin{array}{c} I=\frac{\lambda \theta g\left(x\right)G^{\theta -1}\left(x\right)e^{\lambda }}{{\left\{1-\exp\left[1-e^{\lambda }\right]\right\}}^{t+1}}\overset{\infty }{\underset{z=0}{\sum }}\overset{\infty }{\underset{v=0}{\sum }}\overset{\infty }{\underset{p=0}{\sum }}\frac{{\left(-1\right)}^{z+v+p}}{v!}\left(\begin{array}{c} t\\ z \end{array}\right)\left(\begin{array}{c} v+n-1\\ p \end{array}\right)\\ \times {\left[e^{\lambda }\left(1+z\right)\right]}^{v}e^{-\lambda \left(1+p\right)G^{\theta }\left(x\right)}. \end{array}$$

Finally, we have

(49) $$\begin{array}{c} I=\frac{\lambda e^{\lambda }{\left(q+1\right)}^{-1}}{{\left\{1-\exp\left[1-e^{\lambda }\right]\right\}}^{t+1}}\overset{\infty }{\underset{q=0}{\sum }}\overset{\infty }{\underset{z=0}{\sum }}\overset{\infty }{\underset{v=0}{\sum }}\overset{\infty }{\underset{p=0}{\sum }}\frac{{\left(-1\right)}^{z+v+p+q}}{v!q!}\left(\begin{array}{c} t\\ z \end{array}\right)\left(\begin{array}{c} v+n-1\\ p \end{array}\right){\left[e^{\lambda }\left(1+z\right)\right]}^{v}\times \\ {\left[\lambda \left(1+p\right)\right]}^{q}\theta \left(q+1\right)g\left(x\right)G^{\theta \left(q+1\right)-1}\left(x\right). \end{array}$$

The reduced form becomes

(50) $$\begin{array}{c} f_{Y_{n}^{\left(k\right)}}\left(x\right)=\sum_{q=0}^{\infty }W_{q}h_{\theta \left(q+1\right)}\left(x\right), \end{array}$$

where *h*_*θ*(*q*+1)_(*x*)=*θ*(*q*+1)*g*(*x*)*G*^*θ*(*q*+1)−1^(*x*) and

(51) $$\begin{array}{c} W_{q}=\frac{k!e^{\lambda n}\lambda {\left(q+1\right)}^{-1}}{\left(n-1\right)!}\overset{k-1}{\underset{t=0}{\sum }}\overset{\infty }{\underset{z,v,p=0}{\sum }}\frac{{\left(-1\right)}^{z+v+p+q+t}}{v!q!{\left\{1-\exp\left[1-e^{\lambda }\right]\right\}}^{t+1}}\left(\begin{array}{c} t\\ z \end{array}\right)\left(\begin{array}{c} v+n-1\\ p \end{array}\right)\\ \times \left(\begin{array}{c} k-1\\ t \end{array}\right){\left[e^{\lambda }\left(1+z\right)\right]}^{v}{\left[\lambda \left(1+p\right)\right]}^{q}. \end{array}$$

**Figure 1:**
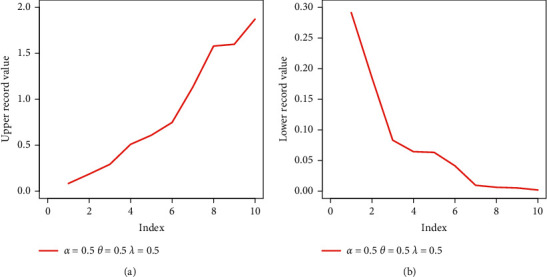
Plot of upper (a) and lower (b) record values of the EBellE model at some parametric values.

A random sample of 50 is generated from the EBellE model using Eq. (23), and then, take *k*=3 and *α*=*β*=*λ*=0.5.  Table 1 shows a random sample of 50 from the EBellE model along with upper *X*_*U*(*n*)_ and lower *X*_*L*(*n*)_ records values. The plot of lower and upper record values is illustrated in Figure 1. The Records package is used in R-Statistical Computing Environment to compute *X*_*U*(*n*)_ and *X*_*L*(*n*)_ records' values.

### 3.10. Stochastic Ordering

Stochastic ordering is another important tool in statistics to define the comparative behaviour specifically in reliability theory. Suppose the two *rvs*, say *X*_1_ and *X*_2_ and under specific circumstance; let us consider that *rv* *X*_1_ is lower than *X*_2_; the readers can refer to the work of Khan et al. [21] for detailed illustration on four stochastic ordering and their well-established relationships.

(52) $$\begin{array}{c} \frac{\text{d}}{\text{d}x}\log\left[\frac{f_{1}\left(x\right)}{f_{2}\left(x\right)}\right]=\theta g\left(x\right)G^{\theta -1}\left(x;\xi \right)\left\{\left[\lambda_{1}-\lambda_{2}\right]+\left[\lambda_{1}e^{\lambda_{1}G^{\theta }\left(x;\;\xi \right)}-\lambda_{2}e^{\lambda_{2}G^{\theta }\left(x;\;\xi \right)}\right]\right\}<0. \end{array}$$

**Theorem 1 .**

*LetX*_1_∼*EBell-G*(*λ*_1_, *θ*; *ξ*)*andX*_2_∼*EBell-G*(*λ*_2_, *θ*; *ξ*)*. Ifα*_1_ ≤ *α*_2_*, thenX*_1_≤_*lr*_*X*_2_:

Proof. First, we have the ratio

(53) $$\begin{array}{c} \frac{f_{1}\left(x\right)}{f_{2}\left(x\right)}=\frac{\lambda_{1}\exp\left\{\lambda_{1}\left[1-G^{\theta }\left(x\right)\right]\right\}\exp\left\{-e^{\lambda_{1}}\left[1-e^{-\lambda_{1}G^{\theta }\left(x\right)}\right]\right\}{\left\{1-\exp\left[1-e^{\lambda_{1}}\right]\right\}}^{-1}}{\lambda_{2}\exp\left\{\lambda_{2}\left[1-G^{\theta }\left(x\right)\right]\right\}\exp\left\{-e^{\lambda_{2}}\left[1-e^{-\lambda_{2}G^{\theta }\left(x\right)}\right]\right\}{\left\{1-\exp\left[1-e^{\lambda_{2}}\right]\right\}}^{-1}}. \end{array}$$

Now, consider

(54) $$\begin{array}{c} \frac{\text{d}}{\text{d}x}\log\left[\frac{f_{1}\left(x\right)}{f_{2}\left(x\right)}\right]=\theta g\left(x\right)G^{\theta -1}\left(x\right)\left[\lambda_{2}-\lambda_{1}\right]+\theta g\left(x\right)G^{\theta -1}\left(x\right)\left[\lambda_{2}e^{\lambda_{2}\left[1-G^{\theta }\left(x\right)\right]}-\lambda_{1}e^{\lambda_{1}\left[1-G^{\theta }\left(x\right)\right]}\right]. \end{array}$$

After simplification, we obtain

(55) $$\begin{array}{c} \frac{\text{d}}{\text{d}x}\log\left[\frac{f_{1}\left(x\right)}{f_{2}\left(x\right)}\right]=\theta g\left(x\right)G^{\theta -1}\left(x\right)\left\{\left[\lambda_{2}-\lambda_{1}\right]+\left[\lambda_{2}e^{\lambda_{2}\left\{1-G^{\theta }\left(x\right)\right\}}-\lambda_{1}e^{\lambda_{1}\left\{1-G^{\theta }\left(x\right)\right\}}\right]\right\}. \end{array}$$

If *λ*_1_ < *λ*_2_, we obtain

(56) $$\begin{array}{c} \frac{\text{d}}{\text{d}x}\log\left[\frac{f_{1}\left(x\right)}{f_{2}\left(x\right)}\right]=\theta g\left(x\right)G^{\theta -1}\left(x\right)\left\{\left[\lambda_{2}-\lambda_{1}\right]+\left[\lambda_{2}e^{\lambda_{2}\left\{1-G^{\theta }\left(x\right)\right\}}-\lambda_{1}e^{\lambda_{1}\left\{1-G^{\theta }\left(x\right)\right\}}\right]\right\}<0. \end{array}$$

Thus, *f*_1_(*x*)/*f*_2_(*x*) is decreasing in *x*, and hence, *X*_1_≤_*lr*_*X*_2_. This completes the proof.

### 3.11. Estimation of Family Parameters

This section is about estimation of the unknown parameters estimation of the EBell-G model by taking into account the popular estimation method known as maximum likelihood estimation (MLE). There are several advantages of MLE over other estimation methods; for instance, the maximum likelihood estimates fulfil the required properties that can be used in constructing confidence intervals as well as maximum likelihood estimates delivering simple approximation very handy while working the finite sample. *ℓ*(.) represent the vector parameters *ϕ*=(*λ*, *θ*, *ξ*)^⊤^; then,

(57) $$\begin{array}{c} \ell \left(\phi \right)=n\;\log\left(\lambda \theta \right)+\overset{\infty }{\underset{i=1}{\sum }}\log\;g\left(x_{i};\xi \right)+\left(\theta -1\right)\overset{\infty }{\underset{i=1}{\sum }}\log\;G\left(x_{i};\xi \right)+\lambda \overset{\infty }{\underset{i=1}{\sum }}\left[1-G^{\theta }\left(x_{i};\xi \right)\right]\\ -n\;\exp\left(\lambda \right)+\overset{\infty }{\underset{i=1}{\sum }}\exp\left\{\lambda \left[1-G^{\theta }\left(x_{i};\xi \right)\right]\right\}-n\;\log\left\{1-\exp\left[1-e^{\lambda }\right]\right\}, \end{array}$$

(58) $$\begin{array}{c} \frac{\partial \ell }{\partial \lambda }=\frac{n}{\lambda }+\overset{\infty }{\underset{i=1}{\sum }}\left[1-G^{\theta }\left(x_{i};\xi \right)\right]-n\;\exp\left(\lambda \right)+\overset{\infty }{\underset{i=1}{\sum }}\exp\left\{\lambda \left[1-G^{\theta }\left(x_{i};\xi \right)\right]\right\}\left[1-G^{\theta }\left(x_{i};\xi \right)\right]\\ -n\;\exp\left(\lambda \right)\frac{\exp\left[1-e^{\lambda }\right]}{\left\{1-\exp\left[1-e^{\lambda }\right]\right\}},\\ \frac{\partial \ell }{\partial \theta }=\frac{n}{\theta }+\overset{\infty }{\underset{i=1}{\sum }}\log\;G\left(x_{i};\xi \right)-\lambda \overset{\infty }{\underset{i=1}{\sum }}G^{\theta }\left(x_{i};\xi \right)\log\left[G\left(x_{i};\xi \right)\right]-\lambda \overset{\infty }{\underset{i=1}{\sum }}\exp\left\{\lambda \left[1-G^{\theta }\left(x_{i};\xi \right)\right]\right\}\\ G^{\theta }\left(x_{i};\xi \right)\log\left[G\left(x_{i};\xi \right)\right],\\ \frac{\partial \ell }{\partial \xi }=\overset{\infty }{\underset{i=1}{\sum }}\frac{g_{i}^{\xi }}{g\left(x_{i};\xi \right)}+\left(\theta -1\right)\overset{\infty }{\underset{i=1}{\sum }}\frac{G_{i}^{\xi }}{G\left(x_{i};\xi \right)}-\lambda \theta \overset{\infty }{\underset{i=1}{\sum }}G^{\theta -1}\left(x_{i};\xi \right)G_{i}^{\xi }\\ -\lambda \theta \overset{\infty }{\underset{i=1}{\sum }}\exp\left\{\lambda \left[1-G^{\theta }\left(x_{i};\xi \right)\right]\right\}G^{\theta -1}\left(x_{i};\xi \right)G_{i}^{\xi }, \end{array}$$

where *g*_*i*_^*ξ*^=*∂*/*∂ξg*(*x*_*i*_; *ξ*) and *G*_*i*_^*ξ*^=*∂*/*∂ξG*(*x*_*i*_; *ξ*) are derivatives of column vectors of the same dimension of *ξ*, and by setting *ϕ*_*λ*_=0, *ϕ*_*θ*_=0, and *ϕ*_*ξ*_=0, the MLEs can be yielded by solving the above equations simultaneously.

**Proposition 7 .**

*A randomly selected sample of sizenis under EBell-G; then, the score vector*(*ϕ*_*λ*_, *ϕ*_*θ*_, *ϕ*_*ξ*_)*is given by*

## 4. Layout of the EBellE Model

**Figure 2:**
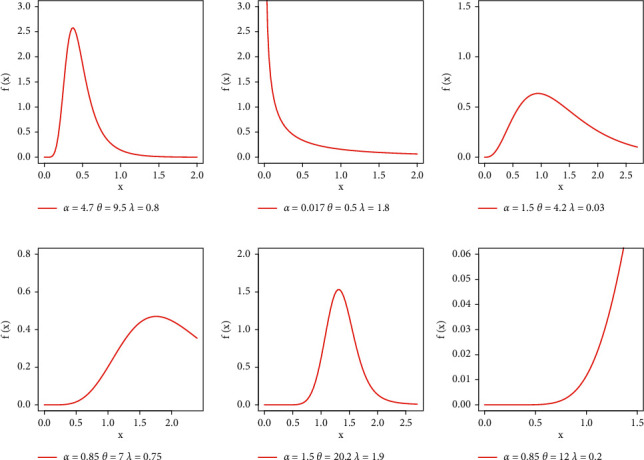
Plots of EBellE density for some parametric values.

**Figure 3:**
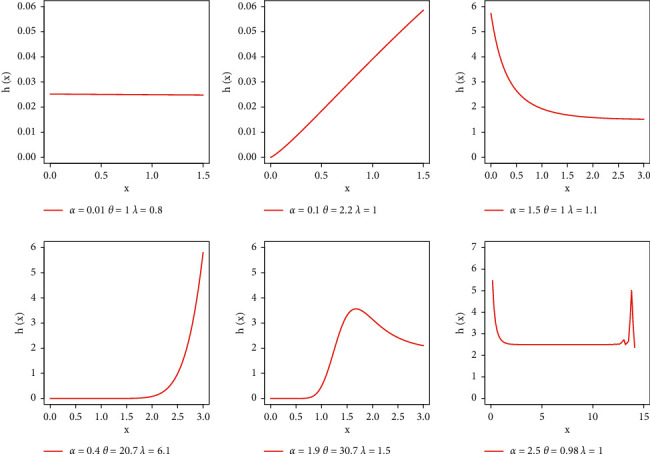
Plots of hazard rate of EBellE for some parametric values.

Due to the closed form solution of many real problems and simplicity, exponential distribution is commonly employed in lifetime testing as well as reliability analysis. However, the exponential distribution failed to yield better fits when hazard rates are nonconstant. However, several studies showed that extended exponential distribution or when it is used as baseline model provides better fits [22–24]. In the present study, we used exponential distribution as a baseline model which yielded flexibility in both pdf and hrf shapes given in Figures 2 and 3, respectively. We now define the EBellE distribution by taking the exponential model as baseline, with the following expression of densities *g*(*x*)=*α*  exp(−*αx*) and *G*(*x*)=1 − exp(−*αx*) for *x* > 0 and *α* > 0, by setting these densities in (7) and (8) yielded the following expression for the proposed EBellE distribution. Then, the cdf and pdf are of the EBellE distribution, respectively.

(59) $$\begin{array}{c} F\left(x\right)=\frac{1-\exp\left\{-e^{\lambda }\left[1-e^{-\lambda {\left[1-e^{-\alpha x}\right]}^{\theta }}\right]\right\}}{\left\{1-\exp\left[1-e^{\lambda }\right]\right\}}, \end{array}$$

(60) $$\begin{array}{c} f\left(x\right)=\lambda \theta \alpha e^{-\alpha x}{\left[1-e^{-\alpha x}\right]}^{\theta -1}\exp\left\{\lambda \left[1-{\left[1-e^{-\alpha x}\right]}^{\theta }\right]\right\} \end{array}$$

**Proposition 8 .**

*LetX*∼*EBellE*(*λ*, *θ*, *α*), *forx* > 0*andλ*, *θ*, *α* > 0; *then*, *its cdf is given by in Eq.* (7):

**Proposition 9 .**

*LetX*∼*EBellE*(*λ*, *θ*, *α*), *forx* > 0*andλ*, *θ*, *α* > 0; *then*, *its pdf is given by in Eq.* (8):

The exponential distribution quantile function becomes *Q*(*u*)=*Q*_*G*_(*z*)=[−1/*α*log(1 − *z*)]; using (9), *z*={1 − *λ*^−1^[log{log{1 − *u*{1 − exp[1 − *e*^*λ*^]}}+exp(*λ*)}]}^*θ*^−1^^. The quantile function of *x* can be expressed as

(61) $$\begin{array}{c} Q\left(u\right)=\left[-\frac{1}{\alpha }\log\left(1-{\left\{1-\lambda^{-1}\left[\log\left\{\log\left\{1-u\left\{1-\exp\left[1-e^{\lambda }\right]\right\}\right\}+\exp\left(\lambda \right)\right\}\right]\right\}}^{\theta^{-1}}\right)\right]. \end{array}$$

The sf and the hrf of the EBellE model can be obtained as

(62) $$\begin{array}{c} s\left(x\right)=\frac{\exp\left\{-e^{\lambda }\left[1-e^{-\lambda {\left[1-e^{-\alpha x}\right]}^{\theta }}\right]\right\}-\exp\left[1-e^{\lambda }\right]}{\left\{1-\exp\left[1-e^{\lambda }\right]\right\}},\\ h\left(x\right)=\frac{\lambda \theta \alpha e^{-\alpha x}{\left[1-e^{-\alpha x}\right]}^{\theta -1}\exp\left\{\lambda \left[1-{\left[1-e^{-\alpha x}\right]}^{\theta }\right]\right\}\exp\left\{-e^{\lambda }\left[1-e^{-\lambda {\left[1-e^{-\alpha x}\right]}^{\theta }}\right]\right\}}{\exp\left\{-e^{\lambda }\left[1-e^{-\lambda {\left[1-e^{-\alpha x}\right]}^{\theta }}\right]\right\}-\exp\left[1-e^{\lambda }\right]}. \end{array}$$

### 4.1. Properties of the EBellE Model

First, we will deduce linear representation of EBellE density to obtain useful properties of that model. By using Eq. (12),

(63) $$\begin{array}{c} f\left(x\right)=\overset{\infty }{\underset{v=0}{\sum }}w_{v}\alpha \theta \left(v+1\right)\exp\left(-\alpha x\right){\left\{1-\exp\left(-\alpha x\right)\right\}}^{\left[\theta \left(v+1\right)-1\right]},\\ {\left\{1-\exp\left(-\alpha x\right)\right\}}^{\left[\theta \left(v+1\right)-1\right]}=\overset{\infty }{\underset{p=0}{\sum }}{\left(-1\right)}^{p}\left(\begin{array}{c} \theta \left(v+1\right)-1\\ p \end{array}\right)\exp\left(-\alpha px\right). \end{array}$$

After applying Eq. (10), it reduces to

(64) $$\begin{array}{c} f\left(x\right)=\sum_{p=0}^{\infty }w_{p}\pi \left[x;\alpha \left(p+1\right)\right], \end{array}$$

where *π*[*x*; *α*(*p*+1)] is a exp-exponential density with *α*(*p*+1) parameter and

(65) $$\begin{array}{c} w_{p}=\sum_{v=0}^{\infty }w_{v}\frac{\theta \left(v+1\right)}{p+1}{\left(-1\right)}^{p}\left(\begin{array}{c} \theta \left(v+1\right)-1\\ p \end{array}\right). \end{array}$$

It is obvious from Eq. (25) that the EBellE density is a linear combination of exponential densities, and therefore, one can obtain several properties using Eq. (25).

#### 4.1.1. The Expression of *r*th Moment

(66) $$\begin{array}{c} \mu_{r}^{\prime }=\sum_{p=0}^{\infty }w_{p}\Gamma \left(r+1\right){\left[\alpha \left(p+1\right)\right]}^{-r}. \end{array}$$

**Proposition 10 .**

*LetX*∼*EBellE*(*λ*, *θ*, *α*), *forx* > 0*andλ*, *θ*, *α* > 0; *then*, *itsrth moment can be written as by taking into account* Eq. (25):

By setting *r*  = 1 yielded the mean of the EBellE model.

#### 4.1.2. The Expression of *s*th Incomplete Moment

(67) $$\begin{array}{c} \varphi_{s}\left(t\right)=\sum_{p=0}^{\infty }w_{p}\gamma \left[s+1,\alpha \left(p+1\right)t\right]{\left[\alpha \left(p+1\right)\right]}^{-s}. \end{array}$$

**Proposition 11 .**

**Figure 4:**
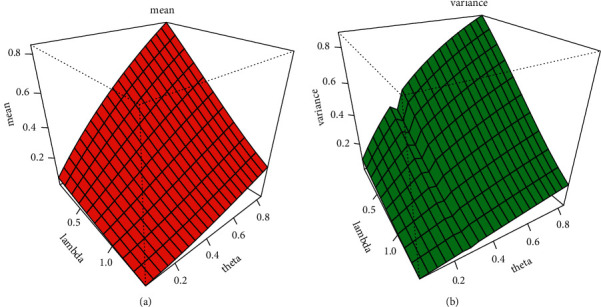
Graphical illustration of mean (a) and variance (b) of the EBellE model.

**Figure 5:**
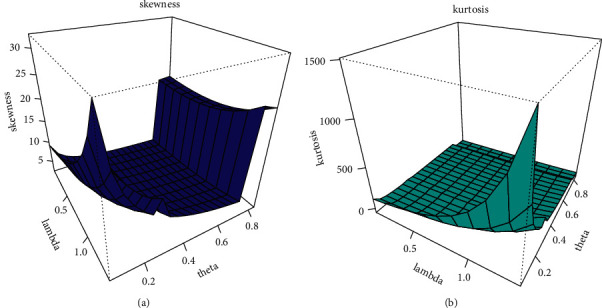
Graphical illustration of skewness (a) and kurtosis (b) of the EBellE model.

**Figure 6:**
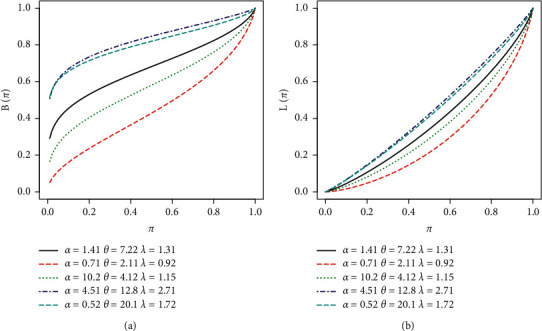
Plot of f Bonferroni (a) and Lorenz (b) curves of EBellE for some parametric values.

*LetX*∼*EBellE*(*λ*, *θ*, *α*), *forx* > 0*andλ*, *θ*, *α* > 0; *then*, *itssth incomplete moment can be written as by taking into account* Eq. (25):

By setting *s*  = 1 yielded the first incomplete moment of the EBellE model.  Table 2 shows the first four raw moments, central moments, coefficient of variation, coefficient of kurtosis, and Pearson's coefficient of skewness for some parametric values. Six different scenarios of parametric values are used in order to compute different measures of dispersion. S-1 = [*α*=2.5, *θ*=1.0, *λ*=0.2], S-2 = [*α*=1.5, *θ*=1.4, *λ*=1.2], S-3 =  [*α*=0.85, *θ*=0.75, *λ*=1.2], S-4 = [*α*=0.85, *θ*=2.5, *λ*=0.2], S-5 = [*α*=4.85, *θ*=0.22, *λ*=0.12], and *S* − 6=[*α*=2.5, *θ*=3.85, *λ*=1]. The following relationship is used to obtain the central moments: *μ*_2_=*μ*_2_′ − (*μ*_1_′)^2^,  *μ*_3_=*μ*_3_′ − 3*μ*_1_′*μ*_2_′+2(*μ*_1_′)^3^, and *μ*_4_=*μ*_4_′ − 4*μ*_3_′*μ*_1_′+6*μ*_2_′(*μ*_1_′)^2^ − 3(*μ*_1_′)^4^. The moment-based measure of skewness and kurtosis is obtained by using *β*_1_=*μ*_3_^2^/*μ*_2_^3^ and *β*_2_=*μ*_4_/*μ*_2_^2^, respectively. Pearson's coefficient of skewness is simply square root of *β*_1_, and coefficient of kurtosis is computed as *β*_2_ − 3. Furthermore, we present the mean, variance, skewness, and kurtosis of EBellE in Figures 4 and 5, respectively, utilizing these results. Some plots of Bonferroni and Lorenz curve are also depicted in Figure 6.

#### 4.1.3. The Expression of *r* th Conditional Moment

From actuarial prospective, conditional moments are important; let EBellE be (*λ*, *θ*, *α*) for *x* > 0 and *λ*, *θ*, *α* > 0; then, its *r* th conditional moment can be written by using Equation (64):

(68) $$\begin{array}{c} E\left(x^{r}|X>t\right)=\frac{1}{{\bar{F}}_{EBellE}\left(t\right)}\int_{t}^{\infty }x^{r}f\left(x\right)\text{d}x,\\ E\left(x^{r}|X>t\right)=\frac{1}{{\bar{F}}_{EBellE}}\overset{\infty }{\underset{p=0}{\sum }}w_{p}\gamma \left[r+1,\alpha \left(p+1\right)t\right]{\left[\alpha \left(p+1\right)\right]}^{-r}. \end{array}$$

#### 4.1.4. Two Expression of MGF

Let *X* ∼ EBellE (*λ*, *θ*, *α*) for *x* > 0 and *λ*, *θ*, *α* > 0; then, its moment generating function by using Wright generalization hypergeometric function is given as

(69) Ψqp=α1,A1,…………,αp,Apβ1,B1,…………,βq,Bq;x=∑n=0∞Πj=1pΓαj+AjnΠj=1qΓβj+Bjnxnn!,

(70) $$\begin{array}{c} M\left(t\right)=a\left(p+1\right)\sum_{p=0}^{\infty }w_{p}\int_{0}^{\infty }\exp\left(tx\right)\exp\left[-a\left(p+1\right)x\right]\text{d}x. \end{array}$$

Consider *I*=∫_0_^*∞*^exp(*tx*)exp[−*a*(*p*+1)*x*]d*x* and exp(*tx*)=∑_*m*=0_^*∞*^*t*^*m*^/*m*!*x*^*m*^; equation (70) is reduced to

(71) $$\begin{array}{c} I=\frac{1}{{\left[a\left(p+1\right)\right]}^{m}}\sum_{m=0}^{\infty }\Gamma \left(m+1\right)\frac{t^{m}}{m!}. \end{array}$$

By using (70), Equation (71) yielded as

(72) I=1ap+1mΨ011,1−;t,Mt=∑p=0∞wp1ap+1mΨ011,1−;t.

The other representation of mgf is given by

(73) $$\begin{array}{c} M\left(t\right)=a\left(p+1\right)\sum_{p=0}^{\infty }w_{p}{\left[a\left(p+1\right)-t\right]}^{-1}. \end{array}$$

#### 4.1.5. Order Statistics

The *s*th moment of order statistic can be obtained by using (41):

(74) $$\begin{array}{c} E\left(X_{i:n}^{s}\right)=\sum_{j=0}^{\infty }Q_{i:n}^{\left(j\right)}\theta \left(j+1\right)\int_{0}^{\infty }x^{s}g\left(x\right)G^{\theta \left(j+1\right)-1}\left(x\right)\text{d}x. \end{array}$$

Simplification yielded the expression of *s*th moments of order statistics:

(75) $$\begin{array}{c} E\left(X_{i:n}^{s}\right)=\sum_{p=0}^{\infty }t_{p}\Gamma \left(s+1\right){\left[\alpha \left(p+1\right)\right]}^{-s}, \end{array}$$

where $t_{p}=\sum_{j=0}^{\infty }{\left(-1\right)}^{p}\left(\begin{array}{c} \theta \left(j+1\right)-1\\ p \end{array}\right)Q_{i:n}^{\left(j\right)}\theta \left(j+1\right)/\left(p+1\right)$.

**Figure 7:**
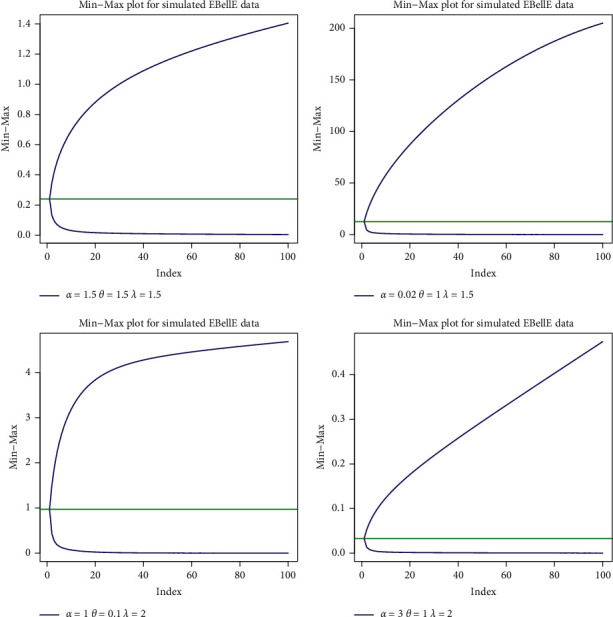
Min-Max plot of order statistics of the EBellE model for some parametric values.

To study the distributional behaviour of the set of observation, we can use minimum and maximum (min-max) plot of the order statistics. Min-max plot depends on extreme order statistics, and it is introduced to capture all information not only about the tails of the distribution but also about the whole distribution of the data.  Figure 7 shows the min and the max order statistics for some parametric values and depends on *E*(*X*_1:*n*_) and *E*(*X*_*n*:*n*_), respectively.

#### 4.1.6. Stochastic Ordering

Let *X* and *Y* be the two *rvs* from EBellE distribution with the assumption previously illustrated in Section 3 given that *λ*_1_ < *λ*_2_, and for *X*_1_≤_*lr*_*X*_2_, *f*_1_(*x*)/*f*_2_(*x*) shall be decreasing in *x* if and the only if the following results holds:

(76) $$\begin{array}{c} \frac{\text{d}}{\text{d}x}\log\left[\frac{f_{1}\left(x\right)}{f_{2}\left(x\right)}\right]=\theta \left[\alpha \;\exp\left(-\alpha x\right)\right]{\left[1-\exp\left(-\alpha x\right)\right]}^{\theta -1}\\ \times \left\{\left[\lambda_{2}-\lambda_{1}\right]+\left[\lambda_{2}e^{\lambda_{2}\left\{1-{\left[1-\exp\left(-\alpha x\right)\right]}^{\theta }\right\}}-\lambda_{1}e^{\lambda_{1}\left\{1-{\left[1-\exp\left(-\alpha x\right)\right]}^{\theta }\right\}}\right]\right\}<0. \end{array}$$

#### 4.1.7. Rényi Entropy

**Figure 8:**
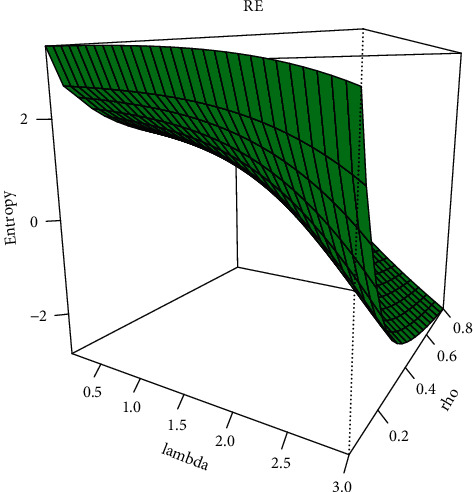
Plot of Rényi entropy of the EBellE model for some parametric values.

The Rényi entropy for the EBellE model by using Eq. (22) given under and *δ* > 0 and *δ* ≠ 1:

(77) $$\begin{array}{c} {\text{I}}_{\delta }\left(X\right)={\left(1-\delta \right)}^{-1}\log\left[\sum_{p=0}^{\infty }Q_{p}{\left[\delta +p\right]}^{-1}\right], \end{array}$$

where $Q_{p}=\alpha^{\delta -1}\sum_{b=0}^{\infty }Q_{b}{\left(-1\right)}^{p}\left(\begin{array}{c} \theta b+\delta \left(\theta -1\right)\\ p \end{array}\right)$, and the graphical demonstration of Rényi entropy of EBellE at varying of the parameters is given in Figure 8.

#### 4.1.8. Reliability

Reliability is an important measure, and several applications are documented in the field of economics, physical science, and engineering. Reliability enables us to determine the failure probability at certain point in a time. Let *X*_1_ and *X*_2_ be the two random variable following the EBellE distribution. The component fails if the applied stress exceeds its strength; if *X*_1_ > *X*_2_, the component will perform satisfactory. Reliability is defined by the following expression. Here, we derive the reliability of the EBellE model when *X*_1_ and *X*_2_ have independent *f*_1_(*x*; *λ*_1_, *θ*, *α*) and *F*_2_(*x*; *λ*_2_, *θ*, *α*) with identical scale (*α*) and shape (*θ*) parameters. The reliability is given by

(78) $$\begin{array}{c} R=\int_{0}^{\infty }F_{2}\left(x\right)f_{1}\left(x\right)\text{d}x. \end{array}$$

By using equations (14) and (15), we get the pdf and the cdf of EBellE, respectively, as follows:

(79) $$\begin{array}{c} f_{1}\left(x;\lambda_{1},\theta ,\alpha \right)=\overset{\infty }{\underset{v=0}{\sum }}w_{v}\left(\lambda_{1}\right)\alpha \theta \left(v+1\right)\exp\left(-\alpha x\right){\left\{1-\exp\left(-\alpha x\right)\right\}}^{\left[\theta \left(v+1\right)-1\right]},\\ F_{2}\left(x;\lambda_{1},\theta ,\alpha \right)=\overset{\infty }{\underset{t=0}{\sum }}w_{t}\left(\lambda_{2}\right){\left\{1-\exp\left(-\alpha x\right)\right\}}^{\left[\theta \left(t+1\right)\right]}. \end{array}$$

Hence,

(80) $$\begin{array}{c} I\left(\alpha ,\beta ,\theta ,v,t\right)=\int_{0}^{\infty }\left[\alpha \;\exp\left(-\alpha x\right){\left\{1-\exp\left(-\alpha x\right)\right\}}^{\left[\theta \left(v+1\right)-1\right]}\right]\\ \times {\left[1-\exp\left(-\alpha x\right)\right]}^{\left[\theta \left(t+1\right)\right]}\text{d}x. \end{array}$$

Therefore,

(81) $$\begin{array}{c} I\left(\alpha ,\beta ,\theta ,v,t\right)=\alpha \sum_{z=0}^{\infty }s_{z}\int_{0}^{\infty }\exp\left[-\alpha \left(1+z\right)x\right]dx. \end{array}$$

By using gamma function, the above expression is reduced to

(82) $$\begin{array}{c} I\left(\alpha ,\beta ,\theta ,v,t\right)=\sum_{z=0}^{\infty }s_{z}{\left[1+z\right]}^{-1}, \end{array}$$

where $s_{z}={\left(-1\right)}^{z}\left(\begin{array}{c} \theta \left[v+t+2\right]-1\\ z \end{array}\right)$.

### 4.2. Estimation

The log-likelihood function for the vector of parameters *ϕ*=(*λ*, *θ*, *α*)^⊤^ for model given in (60) is given by

(83) $$\begin{array}{c} \ell \left(\phi \right)=n\;\log\left(\lambda \theta \alpha \right)-\alpha \overset{\infty }{\underset{i=1}{\sum }}x_{i}+\left(\theta -1\right)\overset{\infty }{\underset{i=1}{\sum }}\log\left[1-e^{-\alpha x_{i}}\right]+\lambda \overset{\infty }{\underset{i=1}{\sum }}\left[1-{\left(1-e^{-\alpha x_{i}}\right)}^{\theta }\right]\\ -n\;\exp\left(\lambda \right)+\overset{\infty }{\underset{i=1}{\sum }}\exp\left\{\lambda \left[1-{\left(1-e^{-\alpha x_{i}}\right)}^{\theta }\right]\right\}-n\;\log\left\{1-\exp\left[1-e^{\lambda }\right]\right\}. \end{array}$$

The components of the score vector *U*(*ϕ*) are

(84) $$\begin{array}{c} U_{\lambda }=\frac{n}{\lambda }+\overset{\infty }{\underset{i=1}{\sum }}\left[1-{\left(1-e^{-\alpha x_{i}}\right)}^{\theta }\right]-n\;\exp\left(\lambda \right)+\overset{\infty }{\underset{i=1}{\sum }}\exp\left\{\lambda \left[1-{\left(1-e^{-\alpha x_{i}}\right)}^{\theta }\right]\right\}\left[1-{\left(1-e^{-\alpha x_{i}}\right)}^{\theta }\right]\\ -n\;\exp\left(\lambda \right)\frac{\exp\left[1-e^{\lambda }\right]}{\left\{1-\exp\left[1-e^{\lambda }\right]\right\}},\\ U_{\theta }=\frac{n}{\theta }+\overset{\infty }{\underset{i=1}{\sum }}\log\left[1-e^{-\alpha x_{i}}\right]-\lambda \overset{\infty }{\underset{i=1}{\sum }}{\left(1-e^{-\alpha x_{i}}\right)}^{\theta }\log\left[1-e^{-\alpha x_{i}}\right]\\ -\lambda \overset{\infty }{\underset{i=1}{\sum }}\exp\left\{\lambda \left[1-{\left(1-e^{-\alpha x_{i}}\right)}^{\theta }\right]\right\}{\left(1-e^{-\alpha x_{i}}\right)}^{\theta }\log\left[1-e^{-\alpha x_{i}}\right],\\ U_{\alpha }=\frac{n}{\alpha }-\overset{\infty }{\underset{i=1}{\sum }}x_{i}+\left(\theta -1\right)\overset{\infty }{\underset{i=1}{\sum }}\frac{x_{i}\exp\left(-\alpha x_{i}\right)}{\left[1-e^{-\alpha \;x_{i}}\right]}\\ -\lambda \theta \overset{\infty }{\underset{i=1}{\sum }}x_{i}\exp\left(-\alpha x_{i}\right){\left(1-e^{-\alpha \;x_{i}}\right)}^{\theta -1}-\lambda \theta \overset{\infty }{\underset{i=1}{\sum }}x_{i}\exp\left(-\alpha x_{i}\right)\exp\left\{\lambda \left[1-{\left(1-e^{-\alpha x_{i}}\right)}^{\theta }\right]\right\}. \end{array}$$

By setting *U*_*λ*_=0, *U*_*θ*_=0 and *U*_*α*_=0, the MLEs can be yielded by solving the above equations simultaneously.

## 5. Actuarial Measures

### 5.1. Value at Risk

Value-at-risk or quantile risk or simply *VaR* is the extensively used as a standard finial market risk measure. It plays an important role in many business decisions; the uncertainty regarding foreign market, commodity price, and government policies can affect significantly firm earnings. The loss portfolio value is specified by the certain degree of confidence say *q*(90%, 95%or99%). VaR of random variable *X* is simply the *q*th quantile of its cdf. If *X* follows the EBellE model; then, its VaR is defined by the following expression:

(85) $$\begin{array}{c} Q\left(u\right)=\left[-\frac{1}{\alpha }\log\left(1-{\left\{1-\lambda^{-1}\left[\log\left\{\log\left\{1-q\left\{1-\exp\left[1-e^{\lambda }\right]\right\}\right\}+\exp\left(\lambda \right)\right\}\right]\right\}}^{\theta^{-1}}\right)\right]. \end{array}$$

### 5.2. Expected Shortfall

The other important financial risk measure is called an expected-shortfall (ES) introduced in [25] and generally considered a better measure than value-at-risk. It is defined by the following expression:

(86) $$\begin{array}{c} {\text{ES}}_{q}\left(x\right)=\frac{1}{q}\int_{0}^{q}VaR_{x}\text{d}x. \end{array}$$

**Figure 9:**
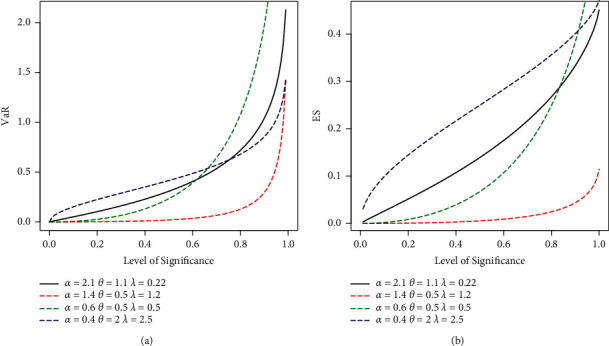
Plot of VaR (a) and ES (b) of EBellE distribution for some parametric values.

For 0 < *q* < 1, using Eq. (85) in Eq. (86), yielded *ES* for EBellE. In Figure 9, the graphical representation of VaR and ES measures for some parameter combinations is presented.

### 5.3. Tail Value at Risk

The problem of risk measurement is one of the most important problems in the risk management. From finance and insurance prospective, Tail value-at-risk (TVaR) or tail conditional expectation or conditional tail expectation is an important measure and define as the expected value of the loss, given the loss is greater than the VaR:

(87) $$\begin{array}{c} \text{TV}aR_{q}\left(x\right)=\frac{1}{1-q}\int_{VaR_{q}}^{\infty }xf\left(x\right)\text{d}x. \end{array}$$

By using (25) in (35) yielded tail value-at-risk as

(88) $$\begin{array}{c} \text{TV}aR_{q}\left(x\right)={\left(1-q\right)}^{-1}\sum_{p=0}^{\infty }w_{p}\gamma \left[2,\alpha \left(p+1\right)VaR_{q}\right]{\left[\alpha \left(p+1\right)\right]}^{-1}. \end{array}$$

### 5.4. Tail Variance

Tail variance (TV) has yet another important risk measure because it considers the variability of the risk along the tail of distribution; it is defined as from the following expression:

(89) $$\begin{array}{c} {\text{TV}}_{q}\left(x\right)=E\left[X^{2}|X>x_{q}\right]-{\left[\text{TV}aR_{q}\right]}^{2}. \end{array}$$

Consider *I*=*E*[*X*^2^*|X* > *x*_*q*_]:

(90) $$\begin{array}{c} I=\text{TV}aR_{q}\left(x\right)=\frac{1}{1-q}\int_{VaR_{q}}^{\infty }x^{2}f\left(x\right)\text{d}x,\\ I=\text{TV}aR_{q}\left(x\right)={\left(1-q\right)}^{-1}\overset{\infty }{\underset{p=0}{\sum }}w_{p}\gamma \left[3,\alpha \left(p+1\right)VaR_{q}\right]{\left[\alpha \left(p+1\right)\right]}^{-2}. \end{array}$$

Using (88) and (90) in (89), we obtain the expression for tail variance for the EBellW model.

### 5.5. Tail Variance Premium

The TVP is the mixture of both central tendency as well as dispersion statistics. It is defined by the following:

(91) $$\begin{array}{c} {\text{TVP}}_{q}\left(X\right)=\text{TV}aR_{q}+\delta {\text{TV}}_{q}, \end{array}$$

where 0 < *δ* < 1. Using expressions (89) and (88) in (91), we obtain the tail variance premium for the EBellW model.

#### 5.5.1. Numerical Illustration of VaR and ES

**Figure 10:**
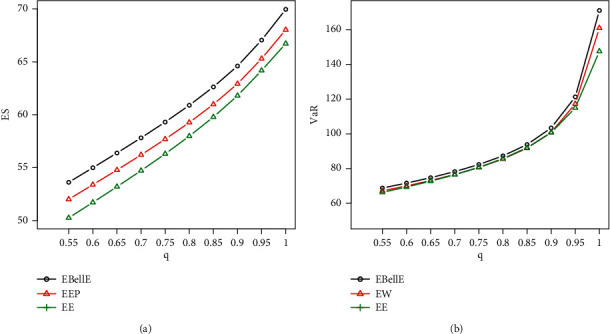
Plot of ES (a) and VaR (b) of EBellE distribution Data-1.

**Figure 11:**
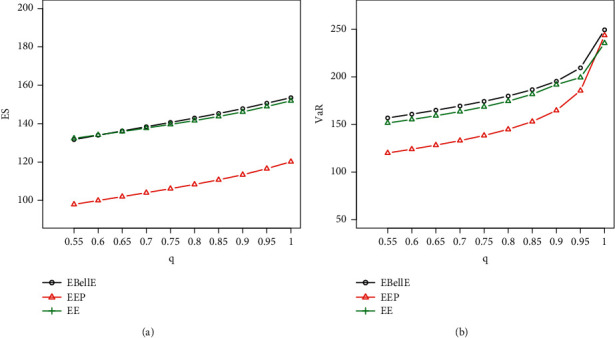
Plot of ES (a) and VaR (b) of EBellE distribution Data-2.

**Figure 12:**
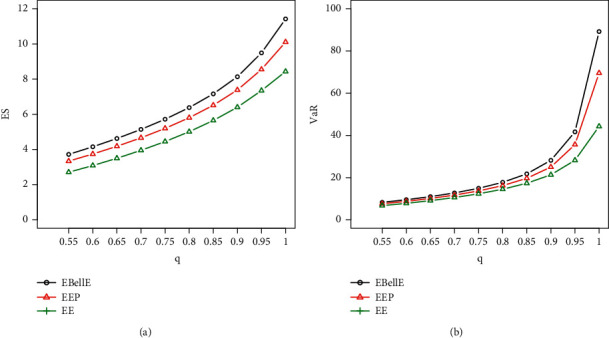
Plot of ES (a) and VaR (b) of EBellE distribution Data-3.

Here, we demonstrate the numerical as well as graphical presentation of the two important risk measures ES and VaR. The comparative study of ES and VaR of the proposed EBellE model with their counterpart exponentiated exponential Poisson (EEP) and exponentiated exponential (EE) model is performed by taking MLEs' estimates of the parameters for the models in all datasets. It is worth emphasising that a model with higher values of the risk measures is said to have a heavier tail.  Table 3 provides the numerical illustration of the ES and VaR for EBellE and EEP and EE model of all three datasets and yielded that the EBellE model has higher values of both the risk measures as compared to their counterpart EEP and EE model. The graphical demonstration of the models from Figures 10–12 also revealed that the proposed model has slightly heavier tail than EEP and EE model. The reader should refer to Chan et al. [26] for detail discussion of VaR and ES and their computation by using an R-Programming Language. A sample of 100 is randomly drawn, and the effect of shape and scale parameters of the proposed models is underlined for both risk measures. Various combinations of the scale and shape parameters are executed I = [*α*=2.1, *θ*=1.1, *λ*=0.22], II = [*α*=1.4, *θ*=0.5, *λ*=1.2], III = [*α*=0.6, *θ*=0.5, *λ*=0.5], and IV = [*α*=0.4, *θ*=2, *λ*=2.5] and change in the curve of VaR and ES are illustrated in Figure 4.

## 6. Designing of GASP under the EBellE Model

Saving time and cost is attributed to the sampling method. Certain quality control checks are implemented either accepting or rejecting a lot under various sampling plans. This section based on the illustration of GASP under the assumption when the lifetime distribution of an item followed a EBellE model with known parameter *λ* and *θ* having cdf in Eq. (96). In a GASP, let a random sample of size *n* be taken and distributed in such a way; that is, *n*=*r* × *g* and *r* items for each group are kept on life testing under predefined time. If the number of failures in each group are higher than the acceptance number *c*, the performed experiment is truncated. The reader is referred to the work of Aslam et al. [27] and Khan and Alqarni [28] for simple illustration of GASP and application to real data. Designing the GASP reduced both the time and cost. Several lifetime traditional and extended models are used [27, 29–32] in designing the GASP by taking into account the quality parameter as mean or median; usually, for skewed distribution, median is preferable [27].

The GASP is simply the extension of ordinary sampling plan (OSP), i.e., the GASP tends to OSP by replacing *r*=1, and thus, *n*=*g* [33].

GASP is based on the following form; first of all, select *g* and allocate predefine *r* items to each group so that the sample of size of the lot will be *n*=*r* × *g*. Secondly, select *c* and *t*_0_ representing the acceptance number and the experiment time, respectively. Thirdly, do experiment simultaneously for *g* groups and record the number of failure for each group. Finally, conclusion is drawn either accepting or rejecting the lot; the lot is accepted if there is no more than *c* failure occurring in each and every group; otherwise, reject a lot. The accepting probability of a lot yielded by the following expression:

(92) $$\begin{array}{c} p_{a\left(p\right)}={\left[\sum_{i=0}^{c}\left(\begin{array}{c} r\\ i \end{array}\right)p^{i}{\left[1-p\right]}^{r-i}\right]}^{g}, \end{array}$$

where the probability that an item in a group fail before *t*_0_ is denoted by *p* and yielded by inserting (61) in (96). Let the lifetime of an item or product follow a EBellE with known parameters *θ* and *λ*, with cdf given for *t* > 0:

(93) $$\begin{array}{c} F\left(t\right)=\frac{1-\exp\left\{-e^{\lambda }\left[1-e^{-\lambda {\left[1-e^{-\alpha t}\right]}^{\theta }}\right]\right\}}{\left\{1-\exp\left[1-e^{\lambda }\right]\right\}}. \end{array}$$

qf of the EBellE model using (61) is given by, and if *p*=0.5 yielded median lifetime distribution for a product or item,

(94) $$\begin{array}{c} m=\left[-\frac{1}{\alpha }\log\left(1-{\left\{1-\lambda^{-1}\left[\log\left\{\log\left\{1-u\left\{1-\exp\left[1-e^{\lambda }\right]\right\}\right\}+\exp\left(\lambda \right)\right\}\right]\right\}}^{1/\theta }\right)\right], \end{array}$$

By taking *η* as follows,

(95) $$\begin{array}{c} \eta =\log\left(1-{\left\{1-\lambda^{-1}\left[\log\left\{\log\left\{1-u\left\{1-\exp\left[1-e^{\lambda }\right]\right\}\right\}+\exp\left(\lambda \right)\right\}\right]\right\}}^{1/\theta }\right). \end{array}$$

Eq. (94) becomes by replacing *η*; henceforth, *α*=−*η*/*m* and *t*=*m*_0_*a*_1_, *m*=−1/*αη*. The ratio of a of product mean lifetime ti and the specified life time *m*/*m*_0_ can be used to express the quality level of product. By replacing *α*=−*η*/*m* and *t*=*m*_0_*a*_1_ in Eq. (96) yielded the probability of failure given by

(96) $$\begin{array}{c} p=\frac{1-\exp\left\{-e^{\lambda }\left[1-e^{-\lambda {\left[1-e^{-\alpha t}\right]}^{\theta }}\right]\right\}}{\left\{1-\exp\left[1-e^{\lambda }\right]\right\}}. \end{array}$$

From Eq. (96), for chosen *θ* and *λ*, *p* can be determined when *a*_1_ and *r*_2_ are specified, where *r*_2_=*m*/*m*_0_. Here, we define the two failure probabilities say *p*_1_ and *p*_2_ corresponding to the consumer risk and producer risk, respectively. For a given specific values of the parameters *θ* and *λ*, *r*_2_, *a*_1_, *β*, and *γ*, we need to evaluate the value of *c* and *g* that satisfy the following two equation simultaneously:

(97) $$\begin{array}{c} p_{a\left(p_{1}|m/m_{0}=r_{1}\right)}={\left[\sum_{i=0}^{c}\left(\begin{array}{c} r\\ i \end{array}\right)p_{1}^{i}{\left[1-p_{1}\right]}^{r-i}\right]}^{g}\leq \beta ,\\ p_{a\left(p_{2}|m/m_{0}=r_{2}\right)}={\left[\sum_{i=0}^{c}\left(\begin{array}{c} r\\ i \end{array}\right)p_{2}^{i}{\left[1-p_{2}\right]}^{r-i}\right]}^{g}\geq 1-\gamma , \end{array}$$

where the mean ratio at consumer's risk and at producer's risk, respectively, is denoted by *r*_1_ and *r*_2_ and the probability of failure to be used in the above expression is as follows:

(98) $$\begin{array}{c} p_{1}=\frac{1-\exp\left\{-e^{\lambda }\left[1-e^{-\lambda {\left[1-e^{a_{1}\eta }\right]}^{\theta }}\right]\right\}}{\left\{1-\exp\left[1-e^{\lambda }\right]\right\}},\\ p_{2}=\frac{1-\exp\left\{-e^{\lambda }\left[1-e^{-\lambda {\left[1-e^{a_{1}\eta {\left(r_{2}\right)}^{-1}}\right]}^{\theta }}\right]\right\}}{\left\{1-\exp\left[1-e^{\lambda }\right]\right\}}. \end{array}$$

From Tables 4 and 5, with *β*=0.25, *a*_1_  = 0.5, and *r*_2_  = 4 and taking *r*  = 5, there are 40 groups or 200 (40 × 5=200); total units are needed for lifetime testing. While on the contrary, significant reduction can be observed in groups or number of units to be tested under the identical circumstances when *r*  = 10; a total of 3 groups or 30 (3 × 10=30) item are needed for life testing. Here, we prefer the group size 10. Similarly, when *β*  = 0.25, *a*_1_  = 1, and *r*_2_  = 4 and taking *r*  = 5, there are 7 groups or 35 (7 × 5=35); total units are needed for life testing. While, on the contrary, in the number of units to be tested under the identical circumstances when *r*  = 10, a total of 2 groups or 20 (2 × 10=20), items are needed for life testing. Here, we prefer the group size 10.

## 7. Simulation Analysis

Simulation analysis is very important tools in statistics and used to determine the performance of estimates over predefine replication at varying sample sizes. So, this section is primarily based on simulation analysis in order to underline the performance parameter estimates of the proposed EBellE model. A simulation process is replicated 1000 times with at varying sample sizes, *n*  = 25, 50, 100, and 500. In Table 6, various combinations of the parameter *α*, *θ*, and *λ* are considered, say scenario I = [*α*=1.5, *θ*=0.3, *λ*=0.5], scenario II =  [*α*=0.15, *θ*=0.5, *λ*=0.5], and scenario III =  [*α*=1, *θ*=0.2, *λ*=0.17]. The finding of the simulation analysis yielded that bias, mean square error (MSE), and average width (AW) of the confidence interval of the parameters reduced as sample size increases. On the contrary, the coverage probabilities (CPs) touch 95% nominal level. So, therefore, the MLEs and their asymptotic results can be used for estimating and constructing confidence intervals for proposed EBellE model parameters. Readers are referred to the work of Sigal et al. [34] for simple but comprehensive way in designing Monte Carlo simulation study by using R-programming language:

(99) $$\begin{array}{c} \text{MSE}\left(\hat{\Theta }\right)=\sum_{r=1}^{1,000}\frac{{\left(\hat{\Theta_{i}}-\Theta \right)}^{2}}{1,000}\text{,}\\ \text{Bias}\left(\hat{\Theta }\right)=\sum_{r=1}^{1,000}\frac{\hat{\Theta_{i}}}{1,000}-\Theta . \end{array}$$

## 8. Practical Implementation of the Proposed EBellE Model

### 8.1. Actuarial Data

**Figure 13:**
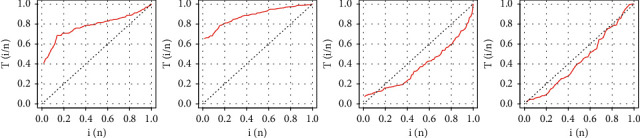
TTT Plots of EBellE of data 1–4.

Here, we demonstrate the flexibility and usefulness of the proposed EBellE model by practical means. Three insurance claim datasets are used; the first two datasets based on unemployment claims from July 2008 to April 2013, reported by the Department of Labour, Licencing, and Regulation, USA. The dataset consists of 21 variables; we used the variable 5 that is new claims filed and variable 12 with total observation for each variable is 58. The dataset was also used by [15]. The third data deal with upheld most frequent complaints such as nonrenewal of insurance, and no fault claims commonly against vehicle insurance company over two-year period as a proportion of their overall business. The dataset was also used by Khan et al. [21]. The descriptive summary of all three datasets is shown in Table 7 and consists of sample size *n*, minimum claim *x*_0_, maximum claim *x*_*n*_, lower *Q*_1_ and upper *Q*_3_, quartile deviations, mean $\bar{x}$, median $\tilde{x}$, standard deviation *σ*, measures of skewness *S*_*k*_, and kurtosis *K*. Total time on test (TTT) plots of the datasets is illustrated in Figure 13, revealing that the first two datasets have increasing hazard rate function, while the third dataset has decreasing (increasing) hazard rate function.

The comparative study is carried out with several modified well-established exponential models, namely, exponentiated exponential Poisson (EEP) [35], alpha power exponentiated exponential (APEE) [15], Transmuted generalized exponential (TGE) [36], gamma exponentiated exponential (GEE) [37], exponential (E), exponentiated exponential (EE), Marshal Olkin exponential (MOE) [38], exponentiated Weibull (EW) [39], odd Weibull exponential (OWE) [19], Weibull (W), Kumaraswamy exponential (KE) [40], beta exponential (BE) [41], Tope Leone exponential (TLE) [42], and Nadarajah Haghigh (NH) [43] distributions.

**Figure 14:**
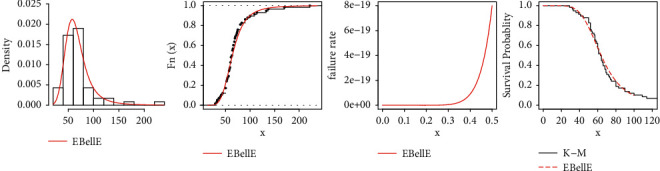
Plots of estimated density, estimated cdf, estimated hrf, and failure rate for data 1.

**Figure 15:**
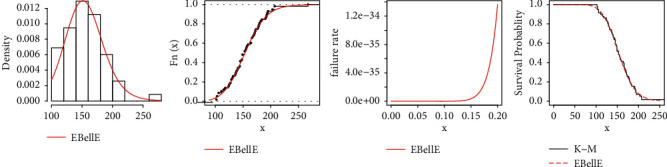
Plots of estimated density, estimated cdf, estimated hrf, and failure rate for data 2.

**Figure 16:**
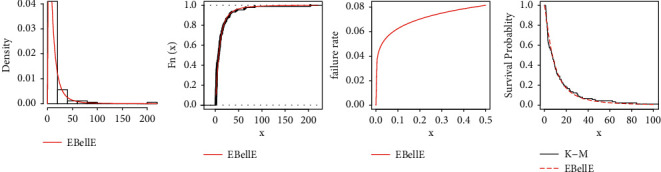
Plots of estimated density, estimated cdf, estimated hrf, and failure rate for data 3.

**Figure 17:**
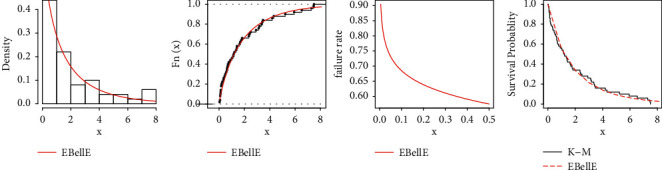
Plots of estimated density, estimated cdf, estimated hrf, and failure rate for data 4.

**Figure 18:**
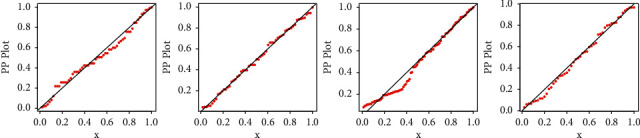
P-P plots of EBellE of data 1–4.

All statistical computational work is carried out using R-programming language.  Table 8 shows the MLEs and standard errors (S.E) of the estimates of the fitted models of the data sets.  Table 9 demonstrated the commonly used well-known model selection information criterion, namely, AIC, CAIC, BIC, and HQIC with important measures including Anderson–Darling (*A*^*∗*^), Cramér–von Mises (*W*^*∗*^), and Kolmogrov–Smirnov (K–S) test and *p* value of all three datasets. The analysis of the datasets revealed the proposed three-parameter EBellE model, outperforming compared to several well established models. A model having higher *p* values and least information criterion and *A*^*∗*^ and *W*^*∗*^, and the K-S value is considered as best models among all other comparative models. TTT plots of the respective datasets are shown in Figure 13. Likewise, plots of the estimated pdf, cdf, hrf, and sf for the four datasets are provided in Figures 14–17. Additionally, PP-plots are presented in Figure 18.

### 8.2. GASP

Recently, Almarashi et al. [29] designed a GASP under Marshall–Olkin–Kumaraswamy exponential distribution by using the data of breaking strength of carbon fibers. The data consist the 50 observed values with mean (1.975) and median (1.190) breaking strength of carbon fibers, respectively. Under the K–S test, the maximum distance between actual and fitted yielded as 0.0681 with *p* value 0.9743 under Marshall–Olkin–Kumaraswamy exponential distribution. We used the same data as data-4, and our proposed three-parameter EBellE model is slightly better fit compared to four-parameter Marshall–Olkin–Kumaraswamy exponential distribution [29] as K-S test as 0.0680 with improved *p* value as 0.9749. The estimated parameters (SEs), namely, $\hat{\alpha }$  = 0.3913 (0.1308), $\hat{\theta }$  = 0.9088 (0.2114), and $\hat{\lambda }$  = 0.3431 (0.5766).  Table 10 shows the GAPS under the EBellE model at MLEs' values showing minimum *g* and *c* when *r*  = 5 and *r*  = 10, with *a*_1_  = 0.5 and 1. The analysis of the data yielded from Table 10, with *β*=0.25, *a*_1_  = 1, and *r*_2_  = 4 and taking *r*  = 5, there are 7 groups or 35 (7 × 5=35); total units are needed for lifetime testing. While, on the contrary, significant reduction can be observed in groups or number of units to be tested under the identical circumstances when *r*  = 10; a total of 2 groups or 20 (2 × 10=20) item are needed for life testing. Here, we prefer the group size as 10. When the true median life increases, the number of groups decreases and the operating characteristics values increases under the EBellE model.

### 8.3. Concluding remarks

We introduced and documented the new flexible family of distributions called exponentiated Bell-G family. We also derived general mathematical properties of the proposed family, namely, linear representation of the density, random variable generation, reliability properties, ordinary moments, generating function, probability weighted moment, entropies, order statistics, reverse order statistics, entropies measures, upper records values, stochastic ordering, and estimation of parameters. We also illustrated the important actuarial measures and design of GASP. We also implemented the new proposed generator to the four real datasets by taking exponential distribution as a special case. The analysis of the data yielded that the new generator is found to be superior compared to their counterparts. [44].

## Figures and Tables

**Table 1:** Upper and lower record values from EBellE generated data.

*n*=50; *k*=3; *α*=*θ*=*λ*=0.5					*X*_*U*(*n*)_	*X*_*L*(*n*)_
0.291681	0.608776	1.872313	0.338539	0.064955	0.083234	0.291681
0.083234	0.399718	0.508271	0.00626	0.042802	0.185290	0.18529
0.18529	0.009471	1.132511	0.022277	0.000141	0.291681	0.083234
0.747575	0.461116	0.118013	2.358966	0.894275	0.509482	0.064443
0.005202	0.120005	0.163145	0.180631	0.052073	0.608776	0.063257
0.041376	0.187393	0.799698	0.983506	0.019917	0.747575	0.041376
0.064443	0.001881	1.599134	1.463232	0.000197	1.132511	0.009471
0.063257	0.059046	0.047385	3.757872	0.259716	1.579419	0.00626
0.509482	0.156173	1.107195	0.719695	0.007713	1.599134	0.005202
0.188353	1.579419	0.118276	0.280922	1.303078	1.872313	0.001881

**Table 2:** Measures of dispersion of the EBellE model for some parametric value.

Measures	S-1	S-2	S-3	S-4	S-5	S-6
*μ*_1_′	0.3591	0.3401	0.3061	1.8307	0.0586	0.4655
*μ*_2_′	0.2722	0.2740	0.4064	5.0198	0.0191	0.3953
*μ*_3_′	0.3181	0.4159	1.1396	18.8237	0.0109	0.4156
*μ*_4_′	0.5022	0.9724	4.8754	90.8662	0.0087	0.5970
*μ*_2_	0.1432	0.1583	0.3127	1.6682	0.0157	0.1786
*μ*_3_	0.1174	0.2150	0.8237	3.5258	0.0079	0.0654
*μ*_4_	0.2061	0.5567	3.6822	20.2673	0.0065	0.1962
*β*_1_	4.6922	11.6396	22.1826	2.6779	16.2771	0.7506
*β*_2_	10.046	22.2016	37.6498	7.2831	26.3259	6.1551
CS	2.1662	3.4117	4.70980	1.6364	4.0345	0.8663
CK	7.0463	19.2016	34.6498	4.2831	23.3259	3.1551

**Table 3:** The detailed summary of ES and VaR of the datasets.

q	Data 1			Data 2			Data 3		
	EBellE	EEP	EE	EBellE	EEP	EE	EBellE	EEP	EE
ES									
0.50	53.614	52.009	50.251	131.699	97.948	132.350	3.7219	3.3382	2.7068
0.60	54.991	53.384	51.710	133.963	99.956	134.106	4.1545	3.7429	3.0869
0.65	56.385	54.776	53.193	136.192	101.971	135.884	4.6243	4.1816	3.4998
0.70	57.816	56.204	54.716	138.409	104.019	137.706	5.1411	4.6628	3.9517
0.75	59.308	57.691	56.301	140.640	106.130	139.596	5.7192	5.1982	4.4508
0.80	60.895	59.268	57.977	142.919	108.345	141.589	6.3803	5.8056	5.0098
0.85	62.629	60.984	59.786	145.287	110.725	143.734	7.1616	6.5147	5.6484
0.90	64.608	62.927	61.801	147.816	113.379	146.116	8.1359	7.3824	6.4018
0.95	67.058	65.299	64.182	150.656	116.561	148.922	9.4909	8.5496	7.3485
0.99	69.963	68.033	66.726	153.517	120.178	151.909	11.4198	10.1086	8.4328
VaR									
0.50	68.734	67.108	66.235	156.895	120.079	151.618	8.3035	7.6248	6.7247
0.60	71.581	69.955	69.327	160.872	124.043	155.273	9.5524	8.7894	7.8348
0.65	74.699	73.063	72.686	165.034	128.320	159.234	11.0126	10.1406	9.1054
0.70	78.208	76.545	76.416	169.485	133.053	163.622	12.7655	11.7457	10.5851
0.75	82.297	80.575	80.673	174.376	138.458	168.619	14.9445	13.7133	12.3490
0.80	87.289	85.450	85.714	179.950	144.904	174.526	17.7899	16.2351	14.5235
0.85	93.819	91.748	92.016	186.649	153.099	181.896	21.7938	19.6942	17.3454
0.90	103.403	100.823	100.649	195.463	164.722	191.974	28.1974	25.0281	21.3472
0.95	121.320	117.303	115.012	209.589	185.624	199.436	41.6908	35.6465	28.2275
0.99	171.119	161.023	147.586	249.567	243.769	235.646	89.1873	69.5513	44.2822

**Table 4:** GASP under the EBellE model, *θ*=1 and *λ*=1.25, showing minimum *g* and c.

*β*	*r*_2_	*r*=5						*r*=10					
		*a*_1_=0.5			*a*_1_=1			*a*_1_=0.5			*a*_1_=1		
		g	c	p(a)	g	c	p(a)	g	c	p(a)	g	c	p(a)
0.25	2	–	–	–	–	–	–	–	–	–	–	–	–
	4	40	3	0.9860	7	3	0.9721	3	3	0.9701	2	4	0.9624
	6	8	2	0.9815	3	2	0.9559	2	2	0.9565	1	3	0.9748
	8	8	2	0.9917	3	2	0.979	2	2	0.9792	1	3	0.9899

0.1	2	–	–	–	–	–	–	–	–	–	–	–	–
	4	66	3	0.9770	12	3	0.9527	5	3	0.9507	5	5	0.9843
	6	12	2	0.9724	12	3	0.9881	5	3	0.9875	2	3	0.9503
	8	12	2	0.9875	4	2	0.9721	3	2	0.9689	2	3	0.9800

0.05	2	–	–	–	–	–	–	–	–	–	–	–	–
	4	85	3	0.9705	95	4	0.9845	17	4	0.9798	7	5	0.9781
	6	16	2	0.9633	15	3	0.9852	7	3	0.9825	2	3	0.9503
	8	16	2	0.9834	5	2	0.9652	3	2	0.9689	2	3	0.9800

0.01	2	–	–	–	–	–	–	–	–	–	–	–	–
	4	131	3	0.9549	146	4	0.9763	26	4	0.9693	10	5	0.9688
	6	131	3	0.9899	23	3	0.9774	10	3	0.9751	5	4	0.9803
	8	24	2	0.9752	7	2	0.9517	10	3	0.9910	3	3	0.9701

Remark: a large sample size in required cells contains hyphens (–).

**Table 5:** GASP under the EBellE model, *θ*=1 and *λ*=1.50, showing minimum *g* and c.

*β*	*r*_2_	*r*=5						*r*=10					
		*a*_1_=0.5			*a*_1_=1			*a*_1_=0.5			*a*_1_=1		
		g	c	p(a)	g	c	p(a)	g	c	p(a)	g	c	p(a)
0.25	2	–	–	–	–	–	–	–	–	–	–	–	–
	4	41	3	0.9866	7	3	0.9735	3	3	0.9718	2	4	0.9644
	6	8	2	0.9824	3	2	0.9578	2	2	0.9584	1	3	0.9761
	8	8	2	0.9921	3	2	0.98	2	2	0.9802	1	3	0.9905

0.1	2	–	–	–	–	–	–	–	–	–	–	–	–
	4	68	3	0.9779	12	3	0.955	5	3	0.9535	5	5	0.9854
	6	13	2	0.9715	12	3	0.9888	5	3	0.9883	2	3	0.9528
	8	13	2	0.9872	4	2	0.9734	3	2	0.9705	2	3	0.9811

0.05	2	–	–	–	–	–	–	–	–	–	–	–	–
	4	88	3	0.9715	95	4	0.9855	17	4	0.9814	7	5	0.9796
	6	16	2	0.9651	15	3	0.9861	7	3	0.9836	2	3	0.9528
	8	16	2	0.9843	5	2	0.9669	3	2	0.9705	2	3	0.9811

0.01	2	–	–	–	–	–	–	–	–	–	–	–	–
	4	135	3	0.9565	146	4	0.9778	26	4	0.9716	10	5	0.9709
	6	135	3	0.9903	23	3	0.9787	10	3	0.9767	5	4	0.9816
	8	25	2	0.9755	7	2	0.954	5	2	0.9513	3	3	0.9718

Remark: a large sample size in required cells contains hyphens (–).

**Table 6:** Biases, MSEs, CPs, and AWs for different scenarios.

Scenario I				Scenario II				Scenario III			
*n*	*α*	*θ*	*λ*	*n*	*α*	*θ*	*λ*	*n*	*α*	*θ*	*λ*
Biases											
25	0.282	0.050	0.152	25	0.006	0.063	0.176	25	0.162	0.067	0.375
50	0.098	0.028	0.105	50	0.002	0.038	0.112	50	0.038	0.048	0.289
100	0.046	0.015	0.050	100	0.010	0.017	0.042	100	−0.007	0.031	0.196
500	0.028	0.005	0.009	500	0.001	0.000	-0.009	500	0.008	0.007	0.043
MSE											
25	1.398	0.010	0.162	25	0.007	0.023	0.198	25	0.669	0.009	0.258
50	0.584	0.005	0.122	50	0.004	0.012	0.145	50	0.204	0.005	0.169
100	0.262	0.002	0.073	100	0.001	0.006	0.094	100	0.001	0.006	0.094
500	0.055	0.001	0.021	500	0.000	0.001	0.029	500	0.000	0.001	0.029
CPs											
25	0.968	0.955	0.982	25	0.972	0.963	0.959	25	0.957	0.938	0.930
50	0.965	0.960	0.980	50	0.976	0.957	0.971	50	0.925	0.927	0.904
100	0.955	0.953	0.969	100	0.940	0.954	0.972	100	0.928	0.927	0.903
500	0.930	0.945	0.950	500	0.940	0.948	0.964	500	0.881	0.965	0.945
AWs											
25	5.001	0.396	2.461	25	0.405	0.627	2.721	25	3.158	0.316	2.375
50	3.123	0.276	1.782	50	0.279	0.455	2.047	50	1.873	0.222	1.751
100	2.078	0.196	1.276	100	0.082	0.327	1.485	100	1.170	0.154	1.271
500	0.887	0.088	0.573	500	0.082	0.149	0.671	500	0.443	0.067	0.607

**Table 7:** Descriptive summary of datasets.

	*n*	*x*_0_	*Q*_1_	$\tilde{x}$	$\bar{x}$	*σ*	*Q*_3_	*x*_*n*_	*S*_*k*_	*K*
Data 1	58	29.000	53.250	63.50	70.670	32.645	74.750	222.00	2.436	10.622
Data 2	58	102.00	133.00	153.00	155.30	31.899	176.00	267.00	0.608	4.0694
Data 3	89	1.0480	2.6160	7.0940	14.079	25.266	15.374	204.17	5.312	37.969

**Table 8:** Estimated parameters and S.Es of insurance data.

Dist.	Parameter	Data 1		Data 2		Data 3	
		Estimates	S.E	Estimates	S.E	Estimates	S.E
EBellE	$\hat{\alpha }$	0.028	0.007	0.0097	0.002	0.022	0.010
	$\hat{\theta }$	10.86	3.442	13.67	3.392	1.174	0.124
	$\hat{\lambda }$	1.34	0.325	2.334	0.345	1.510	0.274

EEP	$\hat{\alpha }$	0.032	0.007	0.015	0.002	0.026	0.011
	$\hat{\beta }$	11.65	3.794	18.01	4.357	1.087	0.119
	$\hat{\lambda }$	3.183	1.410	4.326	1.456	4.400	1.496

APEE	$\hat{\alpha }$	0.032	0.007	0.020	0.007	0.026	0.010
	$\hat{\beta }$	0.040	0.057	0.008	0.024	0.012	0.018
	$\hat{\gamma }$	11.48	3.714	42.11	32.12	1.087	0.119

TGE	$\hat{\alpha }$	0.041	0.006	0.028	0.007	0.044	0.010
	$\hat{\beta }$	0.620	0.262	0.922	0.383	0.772	0.169
	$\hat{\lambda }$	13.63	4.445	80.09	65.40	0.938	0.117

GEE	$\hat{\alpha }$	0.051	0.013	0.132	0.004	0.055	0.010
	$\hat{\beta }$	11.97	8.136	2.819	0.466	1.502	0.738
	$\hat{\lambda }$	1.354	1.139	19.49	0.875	0.557	0.277

EE	$\hat{\alpha }$	0.048	0.006	0.043	0.017	0.063	0.010
	$\hat{\beta }$	16.08	5.250	405.2	903.6	0.837	0.117

MOE	$\hat{\alpha }$	0.066	0.008	0.035	0.003	0.028	0.011
	$\hat{a}$	72.13	41.02	160.9	57.75	0.213	0.106

OWE	$\hat{\alpha }$	0.003	0.001	0.107	0.068	0.003	0.001
	$\hat{a}$	14.28	7.280	0.011	0.005	11.11	2.873
	$\hat{b}$	1.911	0.180	0.245	0.156	0.763	0.055

W	$\hat{\alpha }$	0.585	0.081	0.642	0.083	0.127	0.028
	$\hat{\beta }$	40.33	19.12	24.49	9.451	0.823	0.061

KE	$\hat{\alpha }$	0.073	0.023	0.127	0.003	0.008	0.005
	$\hat{a}$	34.93	27.33	0.081	0.042	0.819	0.074
	$\hat{b}$	0.543	0.247	0.049	0.007	6.133	3.409

BE	$\hat{\alpha }$	0.076	0.025	0.020	0.003	0.015	0.017
	$\hat{a}$	33.00	24.58	34.708	9.900	0.818	0.107
	$\hat{b}$	0.515	0.235	2.146	0.685	3.871	4.198

TLE	$\hat{\alpha }$	0.024	0.003	0.011	0.001	0.031	0.005
	$\hat{a}$	15.98	5.190	17.35	4.216	0.838	0.117

NH	$\hat{\alpha }$	0.003	0.001	0.003	0.001	0.229	0.070
	$\hat{\beta }$	3.900	0.874	2.022	0.347	0.549	0.076
E	$\hat{\alpha }$	0.014	0.002	0.006	0.001	0.071	0.008

**Table 9:** The statistics $\hat{\ell }$, AIC, CAIC, BIC, HQIC, *A*^*∗*^, *W*^*∗*^, K-S, and *p* value for datasets.

Dist.	$-2\hat{\ell }$	AIC	CAIC	BIC	HQIC	*A* ^*∗*^	*W* ^*∗*^	K.S	*P* value
Data 1									
EBellE	264.79	535.57	536.01	541.75	537.98	0.603	0.108	0.096	0.655
EEP	265.31	536.62	537.06	542.80	539.03	0.701	0.127	0.099	0.619
APEE	265.31	536.62	537.06	542.80	539.03	0.703	0.128	0.100	0.610
TGE	266.36	538.71	539.16	544.89	541.12	0.893	0.164	0.102	0.576
GEE	267.65	541.31	541.75	547.49	543.71	1.132	0.209	0.118	0.395
EE	267.49	538.97	539.19	543.09	540.58	1.090	0.201	0.113	0.448
MOE	274.32	552.64	552.85	556.76	554.24	2.218	0.400	0.140	0.204
OWE	572.33	569.93	570.37	576.11	572.33	3.312	0.608	0.187	0.034
W	291.24	586.47	586.69	590.59	588.08	2.065	0.380	0.332	0.000
KE	266.73	539.46	539.90	545.64	541.86	0.991	0.181	0.115	0.430
BE	266.65	539.30	539.74	545.48	541.70	0.975	0.178	0.113	0.448
TLE	267.49	538.97	539.19	543.09	540.58	1.091	0.202	0.113	0.445
NH	295.63	595.27	595.48	599.39	596.87	2.781	0.511	0.371	0.000
E	304.97	611.93	612.01	613.99	612.74	1.755	0.324	0.387	0.000
Data 2									
EBellE	281.25	568.51	568.95	574.69	570.91	0.1887	0.0184	0.0523	0.9973
EEP	284.62	575.24	575.68	581.42	577.64	0.3178	0.0368	0.1089	0.4967
APEE	281.86	569.71	570.15	575.89	572.12	0.4017	0.0497	0.0795	0.8565
TGE	281.56	569.12	569.57	575.30	571.53	0.3841	0.0464	0.0840	0.8078
GEE	281.33	568.66	569.11	574.84	571.07	0.2141	0.0188	0.0545	0.9954
EE	283.74	571.49	571.71	575.61	573.09	0.7022	0.0953	0.1261	0.3146
MOE	291.12	586.24	586.46	590.36	587.85	0.2046	0.0197	0.1687	0.0737
OWE	294.13	594.27	594.71	600.45	596.68	1.3843	0.1875	0.1481	0.1571
W	291.24	586.47	586.69	590.59	588.08	2.0654	0.3799	0.3320	0.0001
KE	357.54	721.08	721.52	727.26	723.49	0.2036	0.0170	0.5291	0.0000
BE	282.53	571.05	571.50	577.24	573.46	0.2880	0.0309	0.0827	0.8220
TLE	293.44	590.88	591.10	595.00	592.49	0.2913	0.0314	0.1756	0.0560
NH	341.65	687.30	687.51	691.42	688.90	0.1982	0.0161	0.4992	0.0001
E	350.62	703.23	703.30	705.29	704.03	0.2091	0.0182	0.4818	0.0001
Data 3									
EBellE	313.42	632.85	633.13	640.31	635.86	1.265	0.185	0.113	0.188
EEP	314.86	635.73	636.01	643.19	638.74	1.410	0.205	0.114	0.182
APEE	314.86	635.73	636.01	643.19	638.74	1.410	0.204	0.114	0.182
TGE	318.91	643.81	644.09	651.28	646.82	1.802	0.262	0.118	0.156
GEE	322.69	651.39	651.67	658.85	654.39	2.191	0.323	0.121	0.134
EE	323.55	651.10	651.24	656.08	653.11	2.271	0.335	0.125	0.114
MOE	316.42	636.83	636.97	641.81	638.84	1.156	0.161	0.127	0.104
OWE	322.55	651.10	651.38	658.57	654.11	1.979	0.286	0.138	0.061
W	320.41	644.82	644.96	649.80	646.83	1.861	0.269	0.126	0.110
KE	321.66	649.32	649.60	656.79	652.33	2.030	0.296	0.118	0.152
BE	323.12	652.24	652.53	659.71	655.25	2.226	0.328	0.117	0.162
TLE	323.55	651.10	651.24	656.08	653.11	2.271	0.335	0.125	0.113
NH	315.90	635.80	635.94	640.78	637.81	1.286	0.182	0.122	0.132
E	324.38	650.76	650.80	653.25	651.76	2.232	0.329	0.162	0.017

**Table 10:** GASP under the EBellE model, $\hat{\theta }=0.9088$ and $\hat{\lambda }=0.3432$, showing minimum *g* and *c* Data 4.

*β*	*r*_2_	*r*=5						*r*=10					
		*a*_1_=0.5			*a*_1_=1			*a*_1_=0.5			*a*_1_=1		
		g	c	p(a)	g	c	p(a)	g	c	p(a)	g	c	p(a)
0.25	2	–	–	–	–	–	–	–	–	–	–	–	–
	4	38	3	0.9808	7	3	0.9668	3	3	0.9585	2	4	0.9547
	6	7	2	0.9766	7	3	0.9908	3	3	0.9880	1	3	0.9678
	8	7	2	0.9887	3	2	0.9726	2	2	0.9686	1	3	0.9860

0.1	2	–	–	–	–	–	–	–	–	–	–	–	–
	4	63	3	0.9683	73	4	0.9850	13	4	0.9763	5	5	0.9800
	6	12	2	0.9602	12	3	0.9842	5	3	0.9800	3	4	0.9836
	8	12	2	0.9806	4	2	0.9636	3	2	0.9533	2	3	0.9721

0.05	2	–	–	–	–	–	–	–	–	–	–	–	–
	4	82	3	0.9590	95	4	0.9806	16	4	0.9709	7	5	0.9722
	6	15	2	0.9505	15	3	0.9804	7	3	0.9722	4	4	0.9782
	8	15	2	0.9758	5	2	0.9547	3	2	0.9533	2	3	0.9721

0.01	2	–	–	–	–	–	–	–	–	–	–	–	–
	4	–	–	–	146	4	0.9703	25	4	0.9549	10	5	0.9605
	6	125	3	0.9840	23	3	0.9700	10	3	0.9605	5	4	0.9728
	8	23	2	0.9632	23	3	0.9883	10	3	0.9842	3	3	0.9585

Remark: a large sample size in required cells contains hyphens (–).

## Data Availability

The data used in the findings of the study are included within the article.

## References

[1] Klugman S. A., Panjer H. H., Willmot G. E. (2012). *Loss Models: From Data to Decisions*.

[2] Cooray K., Ananda M. M. (2005). Modeling actuarial data with a composite lognormal pareto model. *Scandinavian Actuarial Journal*.

[3] Lane M. N. (2000). Pricing risk transfer transactions 1. *ASTIN Bulletin: The Journal of the IAA*.

[4] Vernic R. (2006). Multivariate skew-normal distributions with applications in insurance. *Insurance: Mathematics and Economics*.

[5] Ibragimov M., Ibragimov R., Walden J. (2015). *Heavy-tailed Distributions and Robustness in Economics and Finance*.

[6] Bhati D., Ravi S. (2018). On generalized log-moyal distribution: a new heavy tailed size distribution. *Insurance: Mathematics and Economics*.

[7] Kleiber C., Kotz S. (2003). *Statistical Size Distributions in Economics and Actuarial Sciences*.

[8] Tahir M. H., Nadarajah S. (2015). Parameter induction in continuous univariate distributions: well-established G families. *Anais da Academia Brasileira de Ciências*.

[9] Tahir M. H., Cordeiro G. M. (2016). Compounding of distributions: a survey and new generalized classes. *J Stat Dist Applic*.

[10] Maurya S. K., Nadarajah S. (2020). Poisson generated family of distributions: a review. *Sankhya B*.

[11] Lee C., Famoye F., Alzaatreh A. Y. (2013). Methods for generating families of univariate continuous distributions in the recent decades. *Wiley Interdisciplinary Reviews: Computational Statistics*.

[12] Ahmad Z., Mahmoudi E., Hamedani G. G. (2019). A family of loss distributions with an application to the vehicle insurance loss data. *Pakistan Journal of Statistics and Operation Research*.

[13] Calderin–Ojeda E., Kwok C. F. (2016). Modeling claims data with composite stoppa models. *Scandinavian Actuarial Journal*.

[14] Ahmad Z., Mahmoudi E., Hamedani G., Kharazmi O. (2020). New methods to define heavy-tailed distributions with applications to insurance data. *Journal of Taibah University for Science*.

[15] Afify A. Z., Gemeay A. M., Ibrahim N. A. (2020). The heavy-tailed exponential distribution: risk measures, estimation, and application to actuarial data. *Mathematics*.

[16] Ahmad Z., Mahmoudi E., Hamedani G. (2020). A class of claim distributions: properties, characterizations and applications to insurance claim data. *Communications in Statistics - Theory and Methods*.

[17] Castellares F., Ferrari S. L., Lemonte A. J. (2018). On the bell distribution and its associated regression model for count data. *Applied Mathematical Modelling*.

[18] Bell E. T. (1934). Exponential polynomials. *Annals of Mathematics*.

[19] Bourguignon M., Silva R. B., Cordeiro G. M. (2014). The Weibull–G family of probability distributions. *Journal of Data Science*.

[20] Jamal F., Chesneau C. (2021). The moment properties of order, reversed order and upper record statistics for the power ailamujia distribution. *WSEAS Transactions on Mathematics*.

[21] Khan S., Balogun O. S., Tahir M. H., Almutiry W., Alahmadi A. A. (2021). An alternate generalized odd generalized exponential family with applications to premium data. *Symmetry*.

[22] Gupta R. D., Kundu D. (1999). Theory & methods: generalized exponential distributions. *Australian & New Zealand Journal of Statistics*.

[23] Barreto–Souza W., Cribari–Neto F. (2009). A generalization of the exponential–Poisson distribution. *Statistics & Probability Letters*.

[24] Cancho V. G., Louzada–Neto F., Barriga G. D. (2011). The Poisson–exponential lifetime distribution. *Computational Statistics & Data Analysis*.

[25] Artzner P., Delbaen F., Eber J.-M., Heath D. (1999). Coherent measures of risk. *Mathematical Finance*.

[26] Chan S., Nadarajah S., Afuecheta E. (2016). An r package for value at risk and expected shortfall. *Communications in Statistics - Simulation and Computation*.

[27] Aslam M., Kundu D., Jun C. H., Ahmad M. (2011). Time truncated group acceptance sampling plans for generalized exponential distribution. *Journal of Testing and Evaluation*.

[28] Khan K., Alqarni A. (2020). A group acceptance sampling plan using mean lifetime as a quality parameter for inverse Weibull distribution. *Advances and Applications in Statistics*.

[29] Almarashi A. M., Khan K., Chesneau C., Jamal F. (2021). Group Acceptance sampling plan using marshall–olkin kumaraswamy exponential. *(MOKw–E) Distribution, Processes*.

[30] Srinivasa R. G. (2009). A group acceptance sampling plans for lifetimes following a generalized exponential distribution. *Stochastics and Quality Control*.

[31] Rao G. S. (2009). A group acceptance sampling plans based on truncated life tests for Marshall–Olkin extended Lomax distribution. *Electronic Journal of Applied Statistical Analysis*.

[32] Singh S., Tripathi Y. M. (2017). Acceptance sampling plans for inverse Weibull distribution based on truncated life test. *Life Cycle Reliability and Safety Engineering*.

[33] Aslam M., Shahbaz M. Q. (2007). Economic reliability test plans using the generalized exponential distribution. *Journal of Statistics*.

[34] Sigal M. J., Chalmers R. P. (2016). Play it again: teaching statistics with Monte Carlo simulation. *Journal of Statistics Education*.

[35] Pogany T. K. (2015). The exponentiated exponential Poisson distribution revisited. *Statistics*.

[36] Khan M. S., King R., Hudson I. L. (2017). Transmuted weibull distribution: properties and estimation. *Communications in Statistics - Theory and Methods*.

[37] Ristic M. M., Balakrishnan N. (2012). The gamma-exponentiated exponential distribution. *Journal of Statistical Computation and Simulation*.

[38] Marshall A. W., Olkin I. (1997). A new method for adding a parameter to a family of distributions with application to the exponential and Weibull families. *Biometrika*.

[39] Mudholkar G. S., Srivastava D. K. (1993). Exponentiated weibull family for analyzing bathtub failure-rate data. *IEEE Transactions on Reliability*.

[40] Nadarajah S., Cordeiro G. M., Ortega E. M. (2012). General results for the kumaraswamy g distribution. *Journal of Statistical Computation and Simulation*.

[41] Jones M. C. (2004). Families of distributions arising from distributions of order statistics. *Test*.

[42] Al–Shomrani A., Arif O., Shawky A., Hanif S., Shahbaz M. Q. (2016). Topp-leone family of distributions: some properties and application. *Pakistan Journal of Statistics and Operation Research*.

[43] Nadarajah S., Haghighi F. (2011). An extension of the exponential distribution. *Statistics*.

[44] Afify A. Z., Mohamed O. A. (2020). A new three-parameter exponential distribution with variable shapes for the hazard rate: estimation and applications. *Mathematics*.

